# Cloud-based healthcare framework for real-time anomaly detection and classification of 1-D ECG signals

**DOI:** 10.1371/journal.pone.0279305

**Published:** 2022-12-27

**Authors:** Menaa Nawaz, Jameel Ahmed

**Affiliations:** Department of electrical engineering, Riphah International University, Islamabad, Pakistan; Menoufia University, EGYPT

## Abstract

Real-time data collection and pre-processing have enabled the recognition, realization, and prediction of diseases by extracting and analysing the important features of physiological data. In this research, an intelligent end-to-end system for anomaly detection and classification of raw, one-dimensional (1D) electrocardiogram (ECG) signals is given to assess cardiovascular activity automatically. The acquired raw ECG data is pre-processed carefully before storing it in the cloud, and then deeply analyzed for anomaly detection. A deep learning-based auto-encoder(AE) algorithm is applied for the anomaly detection of 1D ECG time-series signals. As a next step, the implemented system identifies it by a multi-label classification algorithm. To improve the classification accuracy and model robustness the improved feature-engineered parameters of the large and diverse datasets have been incorporated. The training has been done using the amazon web service (AWS) machine learning services and cloud-based storage for a unified solution. Multi-class classification of raw ECG signals is challenging due to a large number of possible label combinations and noise susceptibility. To overcome this problem, a performance comparison of a large set of machine algorithms in terms of classification accuracy is presented on an improved feature-engineered dataset. The proposed system reduces the raw signal size up to 95% using wavelet time scattering features to make it less compute-intensive. The results show that among several state-of-the-art techniques, the long short-term memory (LSTM) method has shown 100% classification accuracy, and an F1 score on the three-class test dataset. The ECG signal anomaly detection algorithm shows 98% accuracy using deep LSTM auto-encoders with a reconstructed error threshold of 0.02 in terms of absolute error loss. Our approach provides performance and predictive improvement with an average mean absolute error loss of 0.0072 for normal signals and 0.078 for anomalous signals.

## Introduction

Physiological signals carry information on the electrical activity taking place in different parts of a human body [1, 2]. Traditionally, this information is analyzed by healthcare workers to evaluate the physiological condition of the patients, and the analysis helps them in decision-making [3]. These decisions have far-reaching consequences on diagnosis, treatment monitoring, drug efficacy tests, and quality of life. Physiological signals such as the electrocardiogram (ECG) carry information on the human heart’s electrical activity, and its analysis helps in understanding the cardiovascular health of the patient [4–7]. Recently, a biometric authentication system has been developed based on a person’s ECG to make it more secure [8]. The recording is performed by conventionally attaching leads to the surface of the human body over specific areas. The graph is then examined by expert healthcare professionals to identify abnormalities or anomalies. The inclusion of computer vision technology and deep learning algorithms specifically has made the process of ECG anomaly detection a matter of a few minutes. The significance of automated detection has been validated in this COVID-19 pandemic when personal visits to a hospital are riskier than the problem itself. This research is an extension of our already published work where we have implemented and analyzed an IoT-based healthcare system, and important features from the biomedical sensor data have been extracted by applying signal processing techniques for anomaly detection [9].

In recent years, the internet of things (IoT) has emerged as one of the most essential communication paradigms [10–12]. IoT can assist the healthcare industry due to its access to information and its ability to ensure connectivity. IoT applications in healthcare have helped people keep track of their medical requirements, such as reminding them of appointments, keeping a check on calorie count, variations in blood pressure, a check on exercises, and many more [13]. The IoT-based healthcare system field comprises sensors, microcontrollers, and other electronic devices. These devices are capable of communicating with each other. The data received from these sensors (which are mostly biomedical sensors) are stored in the cloud for analysis and monitoring performed by medical professionals or attendants. Integrating IoT structures into medical devices advances the quality and service of care for aged patients and children [14]. On-body non-invasive sensors monitor the physiological activities of the human body, and the information is stored and analyzed for the realization, detection, and potential treatment of diseases.

Over the past few years, there have been many advancements in the design of IoT-based systems, which has spurred the use and development of smart systems in various biomedical-related processes. Ultimately, it has supported the improvement of the healthcare system by making it intelligent and manageable. Many examples are describing the effectiveness of smart healthcare, including tracking of patients/biomedical equipment, automatic identification, correct prescription of drugs for patients, and monitoring of physiological parameters of patients in real-time. The rapid increase in cardiovascular disease (CVD) patients has led to the quality study of ECG-acquired patient data and continuous monitoring using the cutting-edge technologies [15–17].

In today’s digital world, artificial intelligence-based techniques especially deep learning algorithms, have influenced every data-driven field and application including augmented reality, video gaming, face detection and recognition, e-health, etc. Deep learning algorithms have great potential to analyze textual data, as well as video data for gathering relevant information [18, 19]. In advanced IoT systems, the challenges are continuously changing and with them, intrusion threats are also rising. Deep learning algorithms can extract the features and identify the anomalies for intrusion detection and classification. In smart home automation and security systems, deep-learning based methods can learn and detect and classify movement patterns for intruder detection. [20, 21]. Recently, in mobile edge computing (MEC) systems, reduced communication latency and offloading computation can be manifested by the reinforcement learning method. In block-chain enabled MEC systems, the reinforcement learning technique is applied to create a secure resource allocation and task offloading in a multi-user system. [19, 22]. In healthcare, they can assist medical staff in the perception, recognition, and prediction of diseases and would also help in decision-making. According to a survey [23], approximately 400,000 people have died in the US due to incorrect diagnoses and decision-making errors of the practitioners. Deep learning algorithms can reduce the diagnosis time and cost and would also help in providing accurate treatment [24, 25]. Time series classification (TSC) of patients’ physiological data helps to recognize and realize anomalies in physiological features by learning and identifying the temporal patterns of the acquired real-time data. TSC of ECG data utilizes the temporal order of the data for analysis, and each time the task involves human cognition [26–28].

Early and accurate detection of a CVD along with its precise treatment can prevent probable deaths. The conventional clinical practice is a tedious and time-consuming process since the CVD diagnosis relies on observing the morphological features of the ECG per-beat recordings, thus an automatic ECG anomaly detection method is desirable [29, 30]. In this research, we have presented an intelligent, automatic 1D ECG anomaly detection and classification method based on AI-based algorithms. The system performs better by improving the feature-engineered parameters of large and diverse datasets. Healthcare data is often in huge volume and to manage long-term batch processing of stored data, an AWS cloud-based architecture is proposed to improve the patient’s data availability, easy data access, and model robustness. Multiclass classification is a challenge due to the contribution of a large and diverse dataset, a wavelet time scattering technique is implemented to decrease the data dimension and make the classification process time-efficient.

This research is an extension of our already published work where we have implemented and analyzed an IoT-based healthcare system, and real-time features from the biomedical sensor data have been extracted by applying signal processing techniques for anomaly detection [9]. The key contributions of this research work include the following:

1. Design and implementation of an end-to-end AWS cloud-based health monitoring framework. The data from on-body sensors have been acquired and stored for real-time monitoring using big data cloud computing techniques. This paper presents an end-to-end AI-edge platform to find the optimized algorithm by comparative investigation of automatic ECG signal classifications.
2. Improved feature set has been developed by combining the carefully crafting hand-engineered features with automatically detected features through AI-based algorithms.
3. The implemented architecture detects anomalies automatically with the help of unsupervised deep LSTM auto-encoders (AEs). LSTM-AE minimizes the loss ratio to decrease the reconstruction error during the training stages on the encoder/decoder.
4. Wavelet time scattering feature extraction is implemented to reduce the signal size to increase the real-time processing speed.

In the remaining part of this article, we present the related study in section II, design, and implementation in section III. This section also includes signal processing of acquired data and time series classification and based on the analysis of signal data, the classification results are discussed in section IV. We then conclude the article in section V.

## Related work

Temporal data have varying abstraction and patterns, and learning the important features for the classification of time series data is a problem that has been addressed by researchers in the past few years. Time series classification groups the temporal data based on extracted features into classes for identification and recognition. In this research, a comparative analysis of more than twenty machine learning is presented. A cloud-based healthcare framework is implemented with improved feature extraction, and extensive machine/deep learning algorithms are applied to learn and train the extracted data for automatic time series classification. Preprocessing and feature extraction through applying signal processing techniques would make the feature extraction process much more reliable and refined. The researchers have invested a good amount of time in analysing signal processing techniques for the construction of structural health monitoring (SHM) for damage detection [31]. A simple Butterworth filter was used to denoise the signal cross-correlation to detect the extent of the damage.

Detection and classification of physiological signals such as ECG for heartbeat diagnosis that has utilized discrete wavelet transform (DWT) and support vector machine (SVM) have resulted in 98.8% classification accuracy [32]. The limitation of such work is usually the vector size. The DWT technique used in this work, lacks the shift-invariance property which affects the performance and accuracy of classification. Other aspects, including the security of industrial IoT-based healthcare, have been explored with the inclusion of encrypted techniques such as watermarking for the prevention of theft. This work doesn’t highlight the efficient data management technique. A large amount of data collected by body sensors in IoT-based healthcare should be managed properly. For this reason, big data analytic techniques have been introduced in healthcare organizations [33–36].

Deep learning algorithms have made their mark in analysing large and varied datasets compared to other classification methods. This is because deep learning algorithms can avail all the available input during the development process [1]. Biomedical signals including electroencephalogram (EEG), ECG, electromyograph (EMG), etc. have been analyzed for this purpose. In another study [14, 37], a healthcare system for critical patients and this system was to be operated on by medical attendants/nurses anywhere. The system has a smartphone application developed to operate with biomedical sensors for acquiring the required data and dedicated health servers. However, a major limitation is the case of GPS/network unavailability and the system’s response time.

The process of classification of a time series involves labeling or assigning a class to the data/time series. As the accessibility of temporal data has increased [38], a large number of algorithms have been proposed to address the classification problem. Generally, TSC can be categorized into feature-based, model-based, and distance-based methods [26, 39–41].

Over the past few years, with advancements in deep neural networks, deep learning algorithms have been introduced to solve time series classification problems with algorithms such as recurrent neural networks (RNNs). The convolutional neural networks (CNNs) have been shown to provide state-of-the-art results on challenging activity recognition tasks with feature engineering and classification tasks in a single algorithm [26, 42, 43]. In deep learning algorithms, the auto-encoder (AE) is an unsupervised way of learning the features of the training data [44]. It reconstructs the input data as an output. Unsupervised AE can learn the important input features by itself by utilizing nonlinear mapping without even labeling the data and then reconstructing the input data. A convolutional auto-encoder is a modified type of AE where a 1D-CNN is used to build a layer that is integrated to extract the important features from raw input data while a 2D-CNN layer is added for image processing [45]. The CNNs have the ability to use the ECG-acquired data as a set of segments to apply 1D CNN, as well as convert it into the image and apply 2D-CNN. To overcome the temporal dependencies, two or more neural networks can be integrated. In [46], CNNs have been used to learn the spatial properties and RNNs have been applied to temporal features. In another research [47], intervals of an ECG signal have been acquired from a 2D image. CNN’s have been implanted for feature extraction while the ECG signal has been decomposed into its components by applying continuous wavelet transform (CWT) for arrhythmia classification. Arrhythmia is the detection of an irregular heartbeat. However, applying CWT for arrhythmia classification can be computationally expensive. In a similar research [48], R-peak has been detected from ECG signal for arrhythmia detection. The classification has been done using CNN and random forest techniques. In [49], the classification accuracy of automatic arrhythmia detection has been improved by combining the temporal (FS1) and frequency domain (FS2) features. The classification has been done using DTCWT and a random forest classifier. The training process in this work depends on the labeling of the ECG data. A hybrid deep CNN algorithm can detect and classify different heartbeats through real-time ECG data for the detection of any abnormality in heartbeats. [50]

In an entropy-based ECG classification, [51], An ECG dataset has been trained in a three neural network-based architecture. First CNN-based, second SincNet based, and third the entropy-induced CNN-based architecture for better computational efficiency. In [52], a solution is given for improving classification accuracy in the case of limited training data available. They have utilized Stockwell transform (ST) technique along with a two-dimensional residual network (2D-ResNet).

The above literature gives deep insights into the contribution of AI technologies in the present healthcare system. However, for long-term batch processing of large volumes of available healthcare data, an end-to-end system is required. The system should be able to store raw data in the big-data cloud, pre-process it for feature extraction and anomaly detection, and an automatic ECG signal classification on the detection of an anomaly, all in a single go. The available literature lacks a multi-function, end-to-end, cloud-based system to deal with continuous temporal changes in a patient’s ECG signal. Moreover, most of the previous research work relies on massive labeled ECG data for training. One of the advantages of this research appears as the multiple steps data processing has been replaced with raw, 1D time series ECG signals to assess cardiovascular activity efficiently. For the time series raw ECG signals, LSTM-based deep learning methods show better accuracy [53], therefore, for automatic anomaly detection of 1D raw ECG time series signals, LSTM-AE is implemented in this research to achieve high accuracy and robustness. For multiclass ECG classification, a large and diverse dataset affects the classification accuracy thus an effective signal reduction technique is required to make the classification process time efficient. Another advantage of this work is that a large volume of healthcare data has been stored in the cloud for long-term batch processing and research analysis. This makes the system more accessible to healthcare experts and researchers.

## Methods and materials

**Fig 1:**
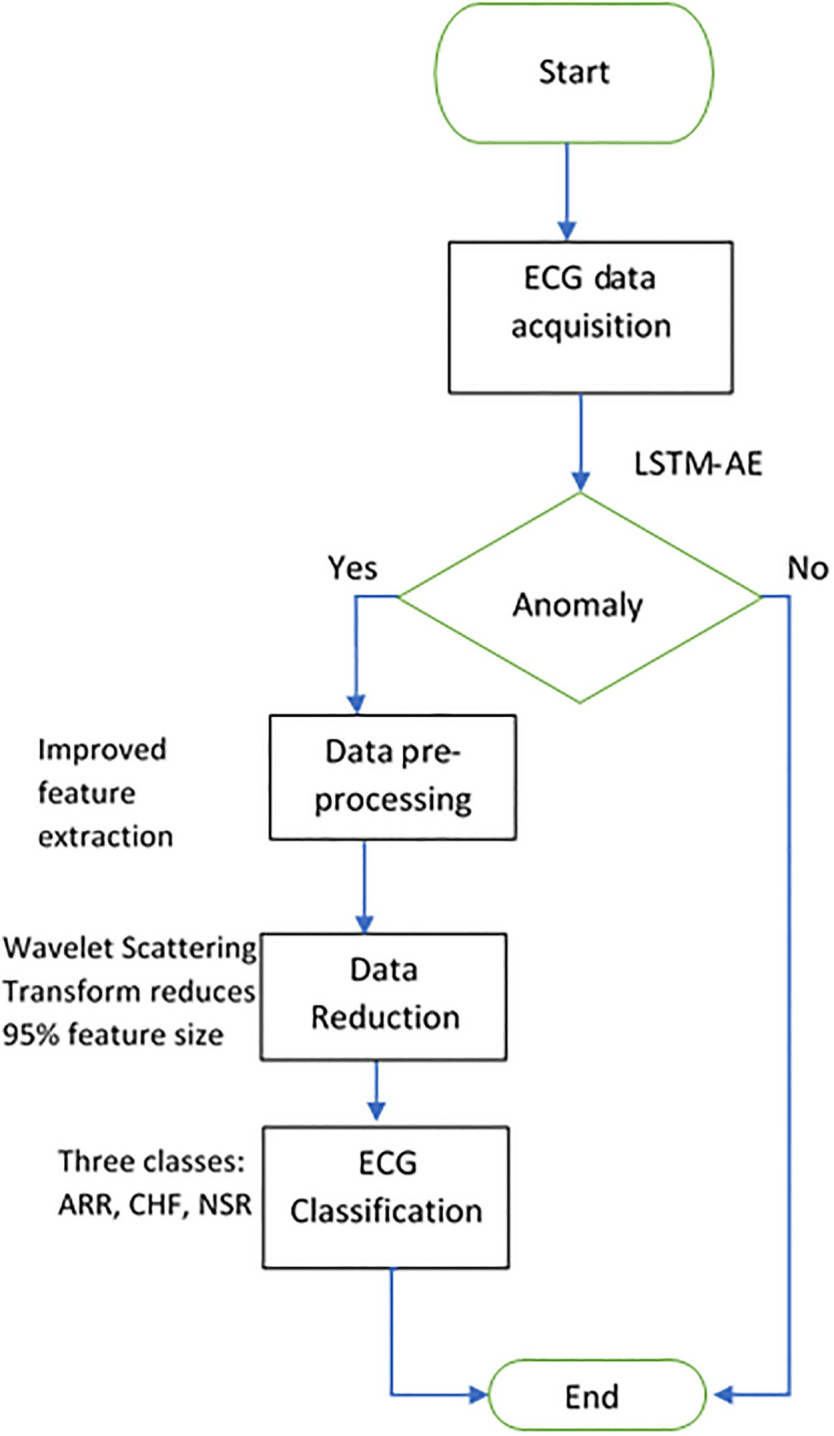
Automatic ECG classification flow.

This work aims to design and implement a cloud-based AI-enabled healthcare system with the ability to combine and enable different, yet complementary technologies. The end-to-end improved automatic ECG classification system in Fig 1, would collect, store, and analyze the raw 1D ECG signals/data about the physiological parameters of a patient and deliver them to a signal processing unit. The ECG data is analyzed for the detection of anomalies, the data is pre-processed for hand-engineered features to make a feature set. Next, the wavelet transform is applied to ECG data to reduce its size to make the system computationally efficient, and then classification takes place in the final step.

Then, finding anomalies in the patient data and sending alerts to a medical professional, helping patients take an active role in managing their health in an end-to-end automatic system.

**Fig 2:**
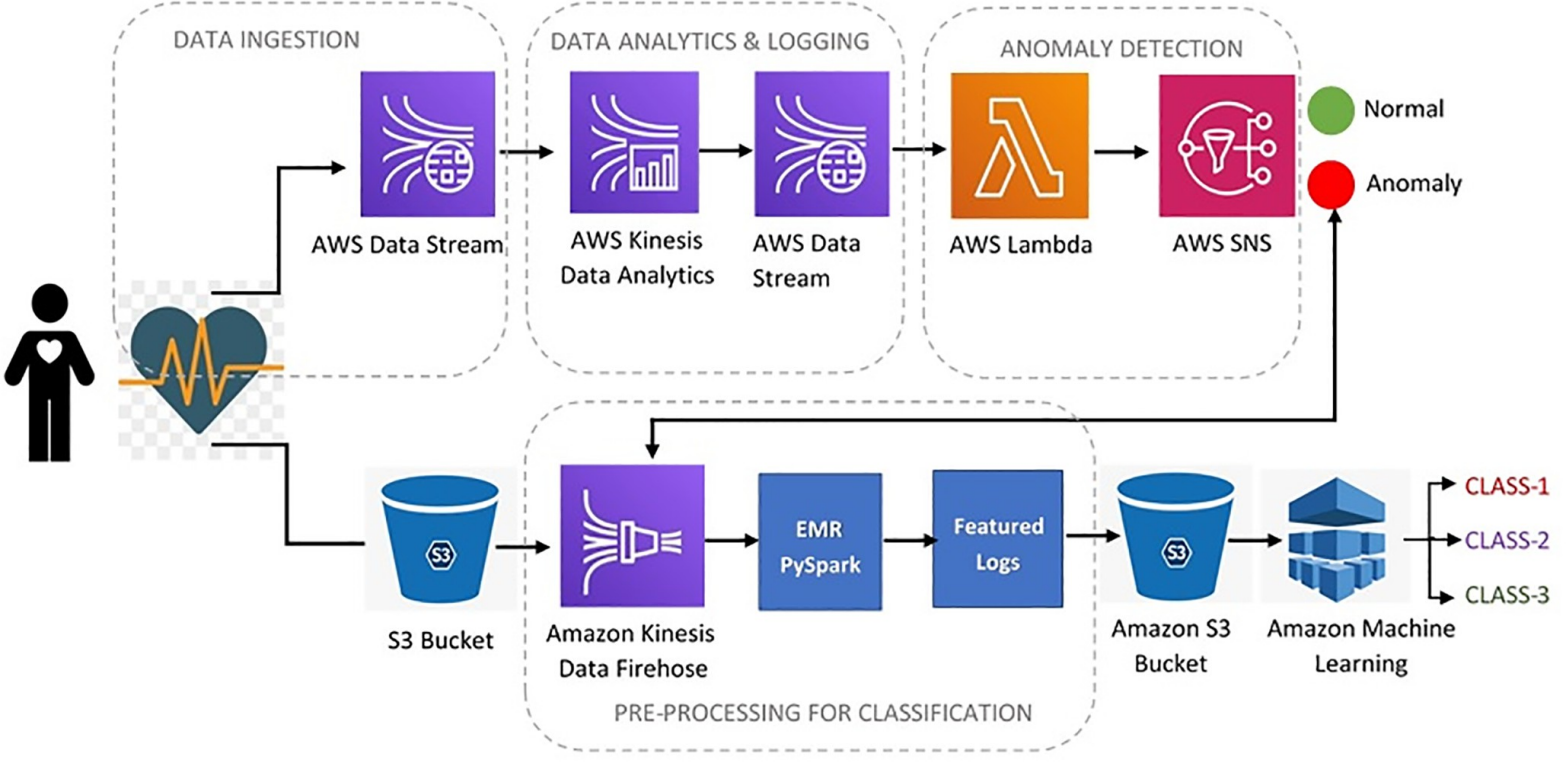
Illustration of the cloud-based healthcare system.

This system comprises raw ECG data, which is often in huge volume, and to manage long-term batch processing of stored data, an AWS [54] cloud-based architecture is proposed. Fig 2 shows the design of the cloud-based healthcare system and signals analysis in the AWS cloud using different data ingestion and analysis tools. Anomaly detection of acquired ECG signals is achieved by a two-step process:

1. Real-time ECG signal anomaly detection.
2. Batch processing for the classification of ECG signals.

In [4] the ECG data is retrieved and processed in LabVIEW for anomaly detection in physiological signals including an ECG for analyzing the heart’s health. The physiological signals used in this research were collected from Physionet [55], the data scarcity has been avoided by merging three different datasets and extracting 162 ECG recordings for three different classes [56–58]. To make it free from baseline wanderings and other motion artifacts, wavelet transform was used. The wavelet transform has its significance due to its better localization properties in both the time and frequency domains [59]. The proposed architecture in the system has used streaming analytics tools of AWS cloud to detect the real-time anomaly existing in the ECG signals. Kinesis data streams [60] are used to extract the data from the ECG logs and feed them to the kinesis data analytics for anomaly detection.

AWS Lambda function is used to detect anomalies in the ECG signal and trigger the Amazon Simple Notification Service (Amazon SNS). Amazon SNS is a fully managed messaging service and communication for application-to-person (A2P) and application-to-application (A2A). In the designed healthcare framework application A2P service is utilized to inform the patient and the doctor-in-the-loop in the case of any anomaly found in the ECG signal. A trigger is also generated in the batch processing layer to further classify the ECG signals to predict the relevant signal class and displayed it to the doctor. This helps in the decision-making process.

For the classification layer, AWS Kinesis Data Firehose is used to feed the logs to the spark clusters for the preprocessing and feature extraction. After extraction of relevant features, these data logs are saved in an amazon simple storage service (Amazon S3) bucket. Amazon SageMaker is deployed on these featured logs to classify the ECG signal classes. Amazon SageMaker is a fully managed machine learning service that helps to easily build and deploy machine learning models in a production-ready hosted environment.

### Data pre-processing

**Fig 3:**
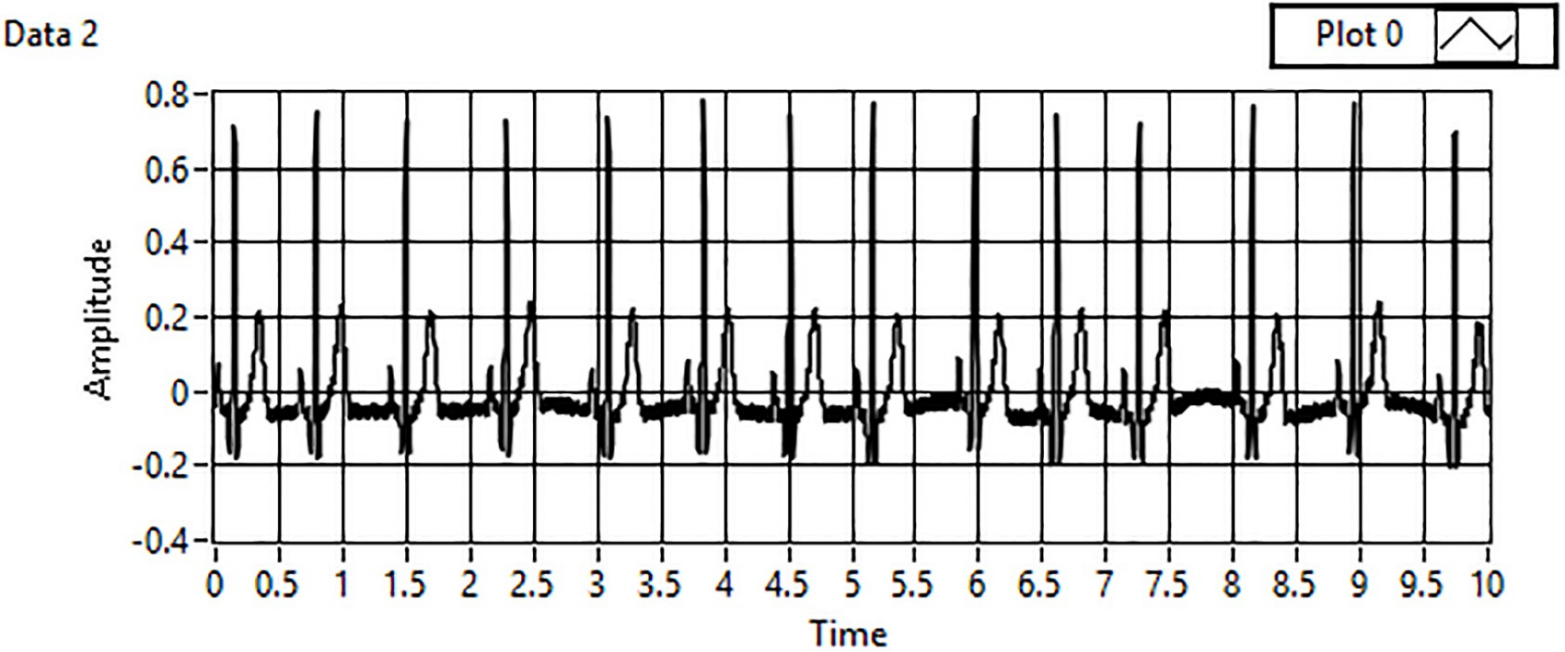
Unfiltered ECG signal.

**Fig 4:**
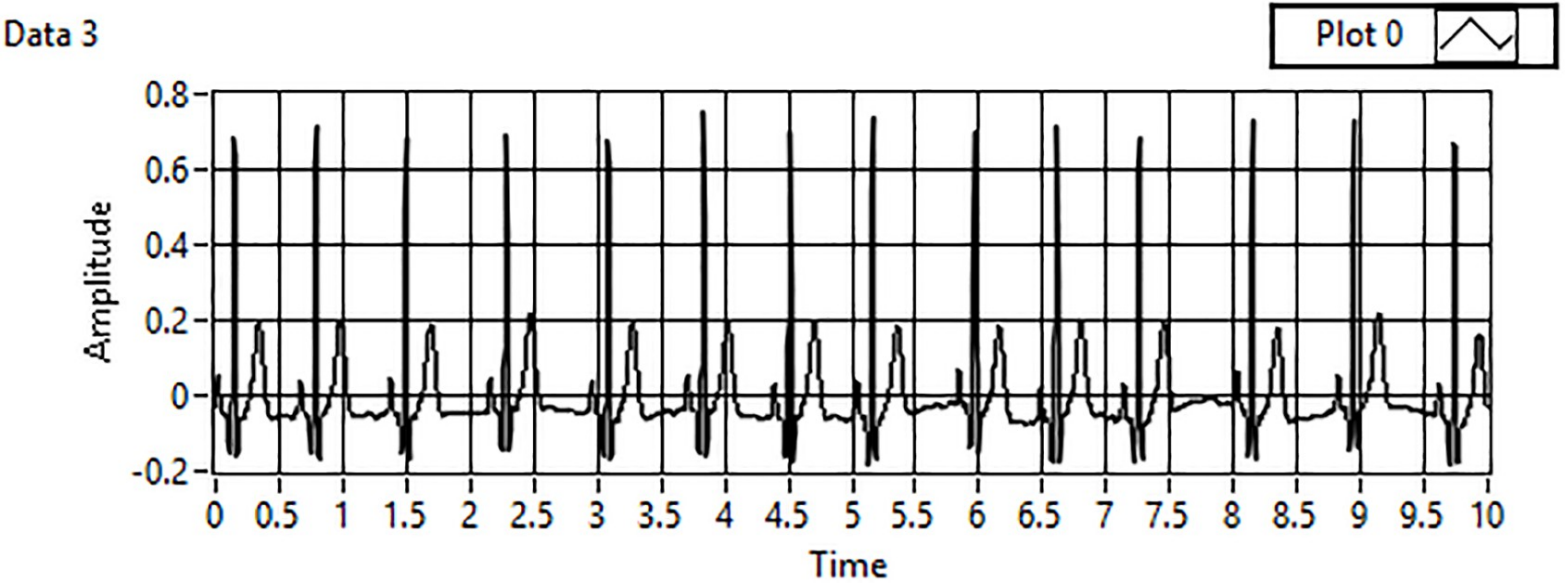
Detrend & denoised ECG signal.

The acquired data are filtered in the first step of the analysis, which includes detrending and denoising of the acquired signals to accurately compare the minimum threshold values already computed into the designed framework. Fig 3 shows the unfiltered ECG signal. A wavelet transform is applied to remove noise or motion artifacts in the ECG signal, as shown in Fig 4.

### Data pipeline

The processed ECG signals are fed into amazon’s Kinesis data streams for creating a real-time pipeline for data analytics. Amazon Kinesis is self-managed, auto-scale Amazon Kinesis data streams have different components like kinesis producers and kinesis consumer’s library which produce and consume data logs. Producers send the data in data streams which are divided into the data at the rate of 1Mb/sec or 1000 messages at write per shard and consumers can consume the data at 2Mb/s across all the users. These features enhance the capability of the healthcare framework by incorporating a central repository to access the data across different locations by different consumers. One important feature of the proposed design is that the data retention period is 24 hours and can be further extended to 7 days if the data is not ingested by the consumers.

### Data reduction

Carefully extracting the important features for training the ECG data is a very significant step in the classification process. Methods such as autoregressive model (AR) [61] and Shannon entropy (SE) [62, 63] have been used for ECG classification in literature. In this research in order to include the temporal response of the signal wavelet scattering transform has been implemented for careful ECG feature extraction. The wavelet transform computes the inner products of a time series signal [64]. Transient ECG signals are present due to the presence of heartbeats. These transients are generally not smooth and are of short duration. Transients are precisely captured by wavelets due to their flexibility in shape and short duration.

For the analysis of the signal f(t), the frequencies have been covered by wavelet function *ψ* and low-pass filter *ϕ*, a group of wavelet indices *A*_*k*_ having octave frequencies. The wavelet transform is for generating the local invariant feature of f is given as:

$$\begin{array}{c} S_{0}f(t)=f\times \phi_{J}\left(t\right) \end{array}$$ (1)

A wavelet transform for the lost higher frequencies in this process is given as:

$$\begin{array}{c} |W_{1}|f=\{S_{0}f\left(t\right),|f\times {\psi_{j}}_{1}\left(t\right){\left|\right\}}_{j_{1}}\in_{A_{1}} \end{array}$$ (2)

By taking the average of wavelet modulus coefficients, the first-order scattering coefficients can be computed as:

$$\begin{array}{c} S_{1}f=\{|f\times \psi_{j}1\left(t\right){\left|\times \phi_{J}\left(t\right)\right\}}_{{j_{1}}_{\in }A_{1}} \end{array}$$ (3)

Complimentary high-frequency components can be computed as:

$$\begin{array}{c} |W_{2}||f\times \psi_{j_{1}}|=\{S_{1}f\left(t\right),||f\times \psi_{j_{1}}\left(t\right)|\times \psi_{j_{2}}\left(t\right){\left|\right\}}_{{j_{2}}_{\in }A_{2}} \end{array}$$ (4)

The second-order coefficients can be computed as:

$$\begin{array}{c} S_{2}f=\{||f\times \psi_{j_{1}}\left(t\right)|\times \psi_{j_{2}}\left(t\right){\left|\times \phi_{J}\left(t\right)\right\}}_{{j_{1}}_{\in }A_{1}};i=1,2 \end{array}$$ (5)

The final scattering matrix for mth-order scattering coefficients is given as:

$$\begin{array}{c} Sf(t)={\left\{S_{m}f(t)\right\}}_{0\leq m\leq l} \end{array}$$ (6)

The repeated application of modulus of the wavelet transform moves the energy contained in the high frequencies of the initial signal towards the low frequencies. At each step of the scattering transform, the energy in the lowest frequency band is output by convolution with *ϕ*_*j*_. The coefficients can be referred to as scattering features and they are down-sampled to reduce the computational complexity of the network.

### Anomaly detection

An autoencoder (AE) is a type of neural network that comes under the category of an unsupervised learning technique, it is capable of learning the best encoding-decoding scheme from the given data. The encoder present in the structure compresses the input data into the latent space while the decoder decompresses the encoded representation into the output layer. The weights in the neural network are adjusted according to the propagated difference error created when the encoded-decoded output is compared with the input data. The setup for a generative model for a latent variable Z is given as *Z* → *f*_*ϕ*_ → *X*, where *f*_*ϕ*_ is a neural network with parameters *ϕ* and *X* be a parametric random variable whose distribution depends on *f*_*ϕ*_(*z*). This model is fit to a dataset *x*_1_, …, *x*_*n*_ and finds the maximum likelihood estimate of the data.

$$\begin{array}{c} L\left(\theta \right)=\text{log}\;\text{Pr}(x_{1},...,x_{n},\theta ) \end{array}$$ (7)

$$\begin{array}{c} L\left(\theta \right)=\sum_{i}\text{log}\;\underset{X}{\text{Pr}}\;(x_{i},\theta ) \end{array}$$ (8)

$$\begin{array}{c} L\left(\theta \right)=\int_{i}\underset{X}{\text{Pr}}\left(x_{i}|Z=z;\theta \right){\text{Pr}}_{Z}\left(z\right)dz \end{array}$$ (9)

Where *i* is all the elements in the vector. An AE is simply a feed-forward deep-neural network where instead of predicting an output as the class label, we are reconstructing the input signal *x*. So, to train an auto-encoder we need to define a loss function that would compare the input signal *x* and reconstructed signal $\hat{x}$. The loss function for real-valued is given as:

$$\begin{array}{c} l\left(f(x)\right)=\frac{1}{2}\sum_{i}{\left(\hat{x_{i}}-x_{i}\right)}^{2} \end{array}$$ (10)

### ECG classification

This research work presents an extensive comparative analysis of artificial intelligence (AI) based algorithms including both machine learning and deep learning algorithms, to find the best possible option for the ECG classification model into three classes: arrhythmia (ARR), congestive heart failure (CHF), and normal sinus rhythm (NSR). The signal length of each of these samples is 65,536 samples long, and we have 162 records of ECG data. A typical, easy way to train machine learning models is to directly feed our raw ECG signals in a machine learning classification algorithm, which would give us outputs such as the ARR and CHF. However, unfortunately, this approach does not work, probably because the signals are lengthy and the features of the signals change very rapidly with time, which the machine learning model is unable to interpret. We have added the step of feature engineering to extract the features from the ECG signals and use those features to train our machine learning algorithms, which helps build this model with higher accuracy. The feature engineering step has numerous advantages and numerous aspects because it reduces the dimensions of the input data. Then, feature selection is also a part of feature engineering, which allows us to select the features that are more prominent or have a greater effect on training the machine learning models, and we can eliminate the other features, which are probably adding unwanted signals to the input data set.

#### K-Nearest neighbor (KNN) method

The k-nearest neighbor (KNN) classification method is a machine learning algorithm that works without a training stage step. The classification tasks are performed by calculating the distance between the test sample and all training samples as a first step, to obtain its nearest neighbors.

This method includes two parts: a) determining k-close neighbors, b) determining class type using these close neighbors. Consider the training data space D described as *D* = *X*_1_, *X*_2_, *X*_3_, …… *X*_*n*_, Which includes n samples, and each sample *X*_*i*_ is defined as: *X*_*i*_ = *x*_*i*1_, *x*_*i*2_, ….., *x*_*if*_.

Considering three different classes in the training data, to determine the particular class $\hat{x}$ in the given data, we need to know its distance from each element in the data present in space D. One of the most common methods for calculating the distance between $\hat{x}$ and *x*_*i*_ is the Euclidean distance criteria. Euclidean distance is defined as the given equation.

$$\begin{array}{c} d=\sqrt{{\left(x_{i1}-\hat{x_{1}}\right)}^{2}+{\left(x_{i2}-\hat{x_{2}}\right)}^{2}+\ldots +{\left(x_{\text{if}}-\hat{x_{f}}\right)}^{2}} \end{array}$$ (11)

#### Support vector machine (SVM) method

One of the important machine learning algorithms for the classification of pattern recognition is the support vector machine (SVM) method. An SVM is a supervised machine-learning algorithm that can be employed for both classification and regression purposes. The SVM algorithm has been used widely while dealing with real-time data problems. Support vector machines are binary classifiers. However, in real cases, data is to be classified into more than 2 classes. This is done by using multi-class SVM. This can be done by using the one-against-one (OAO) approach. According to that approach, for a 3-class classifier as required in our case, (*c*(*c* − 1))/2 number of SVMs are trained. In our case of multi-class SVM, c is 3.

#### RNN-LSTM method

**Fig 5:**
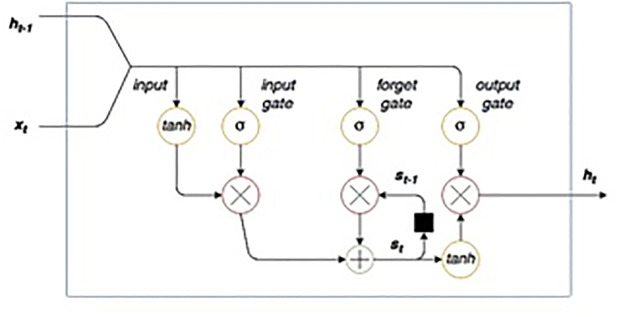
LSTM cell diagram [66].

The long short-term memory (LSTM) network is a type of recurrent neural network (RNN). Instead of mapping inputs to outputs alone, RNN contains cycles of mapping function for the inputs over the previous time steps to an output. Training of RNN has been a challenge to the researchers due to either vanishing gradients or exploding gradients [65]. In vanishing gradients, weight updating resulted in smaller weights and have no further impacts. Similarly, exploding gradients have weights updating resulting in overflow. The architecture of the LSTM memory cell is shown in Fig 5 LSTM–RNN architecture models a non-linear dynamic system by mapping the input sequence to the output sequence. The input vector at sampling interval *t* is concatenated with the LSTM cell hidden state *h*(*t* − 1), state of the previous sample. This input is passed through the tan*h* activation function and at the second step combined input is passed via the input gate which is a layer of sigmoid function which controls the input flow by making input gate weights close to 0 or 1. In the next step, data flows in the cell through the internal state’s t0 and forget gate which also helps to drop unwanted input. Input data is added to the one-step lagged *s*(*t* − 1) recursively.

Finally, the output state is reversed by the tan*h* function which is controlled by the output gate. In the LSTM memory cell, the input, output and forget gates allow the LSTM to forget or write new information to the memory cell. Mathematical equations of the memory cell are given below:

$$\begin{array}{c} i_{k}=\eta (W_{\text{xi}}x_{k}+W_{\text{hi}}h_{k-1}+b_{i}) \end{array}$$ (12)

$$\begin{array}{c} i_{k}=\eta (W_{\text{xf}}x_{k}+W_{\text{hf}}h_{k-1}+b_{f}) \end{array}$$ (13)

$$\begin{array}{c} c_{k}=f_{k}c_{k-1}+i_{k}\text{tanh}(W_{\text{xc}}x_{k}+W_{\text{hc}}h_{k-1}+b_{c}) \end{array}$$ (14)

$$\begin{array}{c} o_{k}=\eta (W_{\text{xo}}x_{k}+W_{\text{ho}}h_{k-1}+b_{o}) \end{array}$$ (15)

$$\begin{array}{c} h_{k}=o_{k}\text{tanh}c_{k} \end{array}$$ (16)

Where *o, i, f, c* are the output, input, forget gates, and memory cell of the network.

The architecture of a simple LSTM network for classification problems starts with a sequence input layer followed by an LSTM layer.

#### Kinesis data analytics

It is used for real-time data analytics and data streaming. In the proposed framework, it is used for anomaly detection in ECG signals. Once the anomaly is detected, the corresponding ECG signals of the 10 secs window are saved/streamed into the kinesis data firehose for further classification of ECG signals. These logs are saved in the S3 bucket for future reference and data repository.

## Results and discussion

### Input data

**Fig 6:**
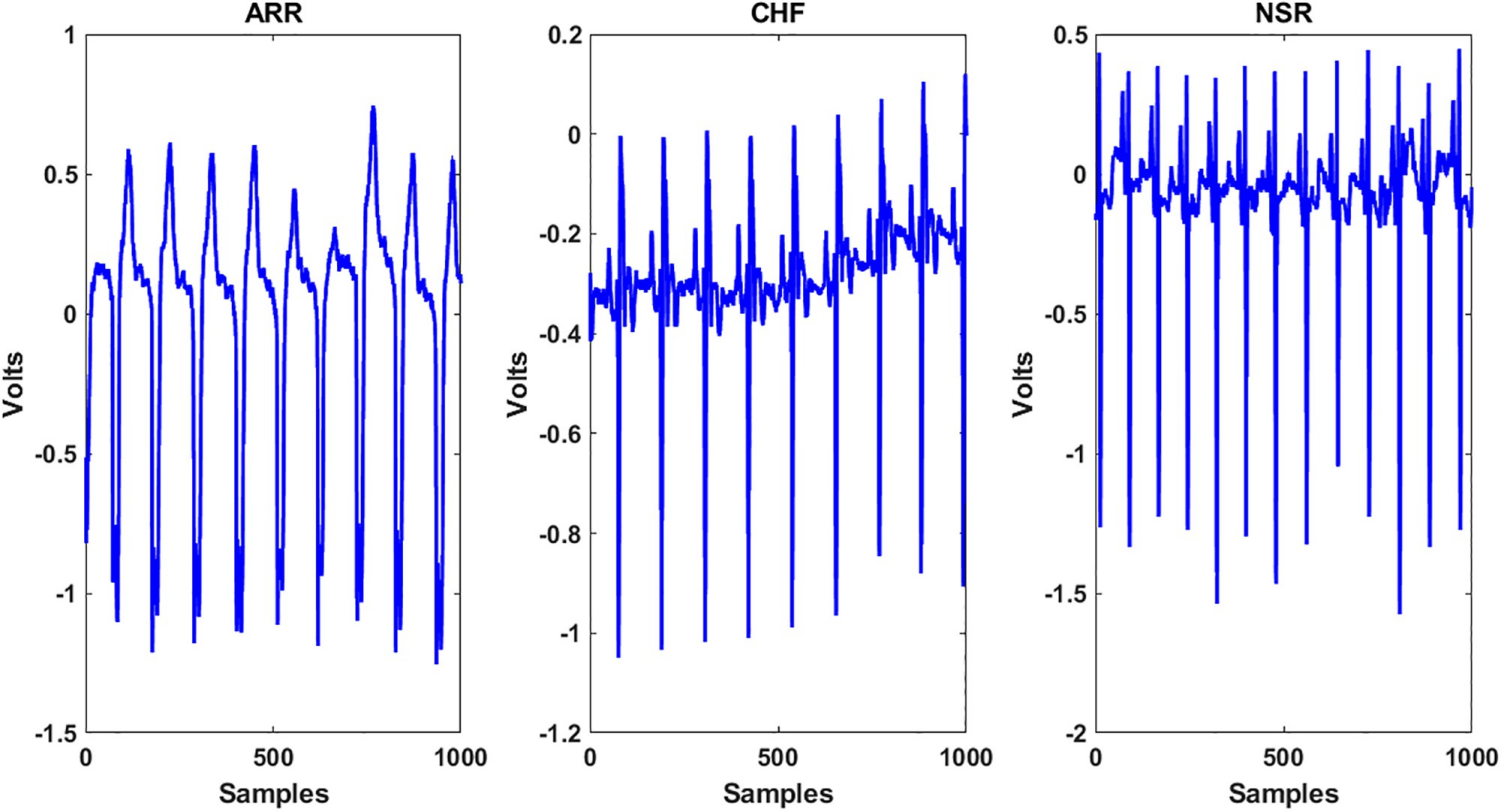
Signal plot of each class.

The physiological signals used in this research were collected from Physionet [55]. Three datasets are used in this research: the MIT-BIH arrhythmia database [56], MIT-BIH normal sinus rhythm database [57], and BIDMC congestive heart failure database [58]. Data have three classes: ARR, CHF, and NSR, with a total of 162 records. Training data are structured into an array of two fields with data and labels. The data field is a 162x65536 matrix, where each row is a training recording sampled at 128 Hz. The sample Plot of each class is shown in Fig 6.

### Improved feature engineering

**Fig 7:**
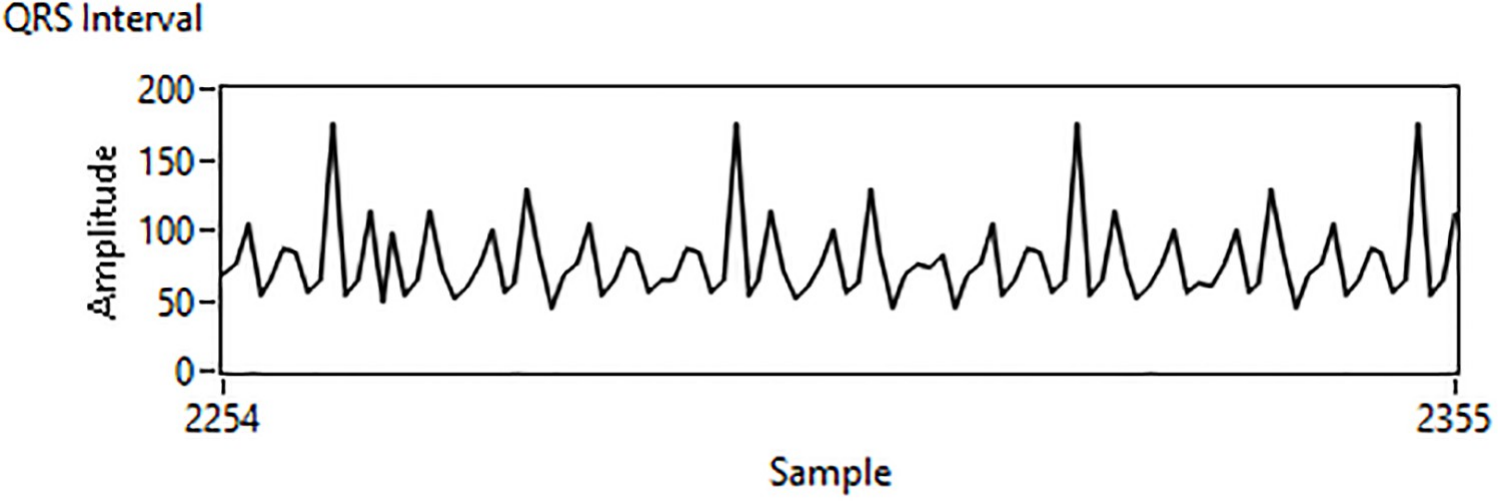
Peak and interval detection in ECG Signal.

**Fig 8:**
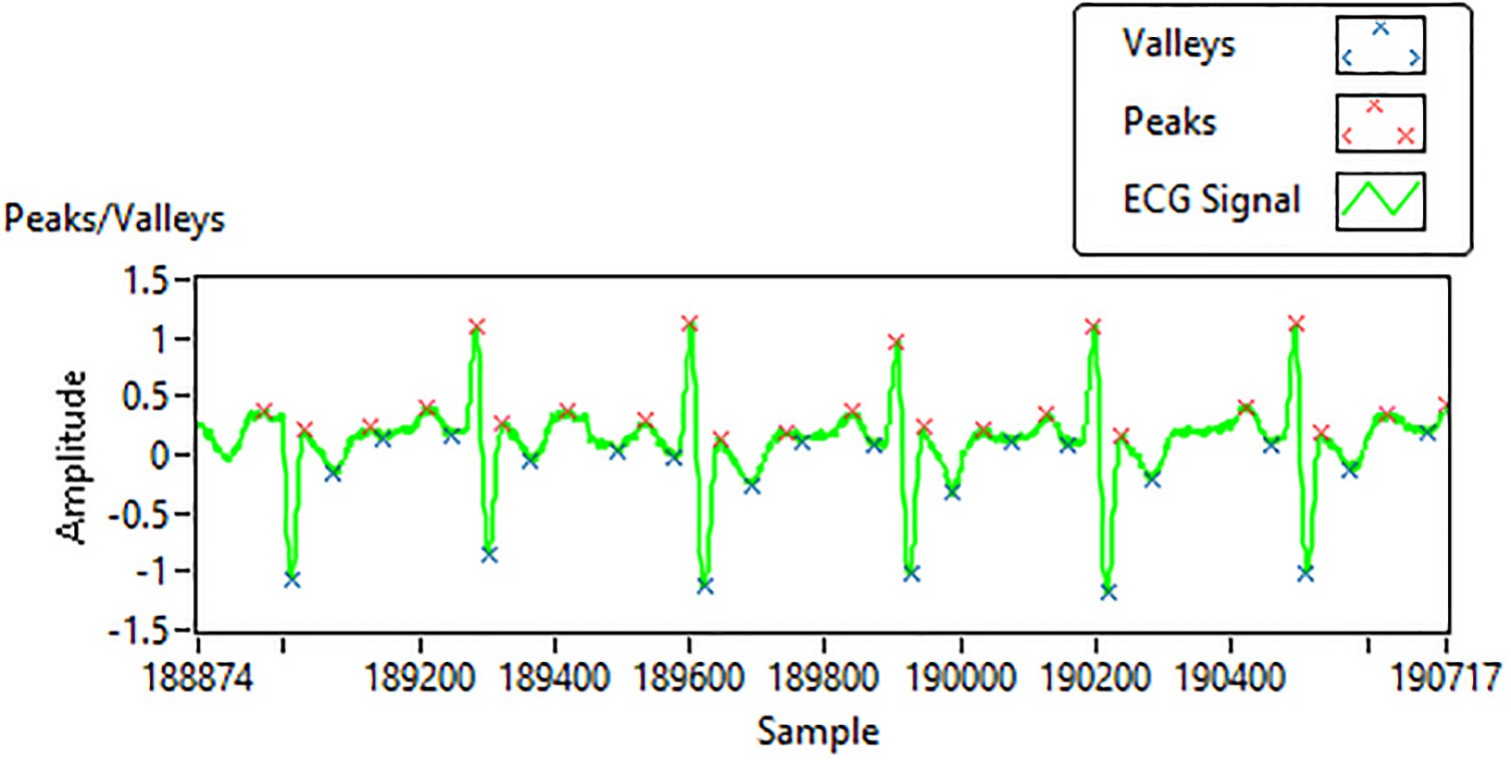
Detection of QRS interval.

In this research, carefully crafted hand-engineered features have been combined with automatically detected features from machine learning (ML) and deep learning (DL) algorithms to form a high-dimensional improved feature set. Hand-crafted features have been selected on the basis of standard clinical mean and median values of ARR, NSR, and CHF. This feature set is also helpful in generating early signs of an anomaly to the doctor-in-loop for timely treatment. The decomposition of a normal ECG signal waveform comprises a P wave, QRS complex, and T wave. These are the important parameters we located during the analysis of the detected ECG signals. To understand the cycle for better analysis, the P wave relates to the heart’s atrial depolarization, and the T wave corresponds to the heart’s ventricular repolarization. The interval between two consecutive R waves is known as the R-R interval, the presence of an arrhythmic heartbeat would impact the RR interval length. The QRS wave is the combination of three deflections and it represents depolarization and contraction of ventricles. The R-R interval and QRS interval as shown in Figs 7 and 8, serve as important indicators of various heart diseases, such as arrhythmia.

In Table 1 the extracted intervals and peak values have been shown, which are compared to threshold normal values (stored manually) for generating medical emergency alerts. The PR, QT, and ST intervals discussed in Table 1 indicate the depolarization of ventricles in segments. The ECG data are received via a biosignal acquisition device and stored in the cloud. If the variation in LabVIEW-detected particular intervals is large, irregular ECG is detected.

**Table 1:** Comparative analysis of ECG feature values and their standard deviation.

Extracted features	Normal values	Tachycardia detection	Hyperglycemia detection
Heart rate mean (bpm)	78.06	119.89	84.07
Heart rate std.(bpm)	0.84	0.98	0.85
QRS amplitude mean (mv)	0.86	1.182	0.635
QRS amplitude std. (mv)	0.022	0.011	0.018
QRS time mean (s)	0.062	0.145	0.056
QRS time std. (s)	0.002	0.0078	0.002
PR interval mean(s)	0.13	0.13	0.141
PR interval std. (s)	0.009	0.023	0.012
QT interval mean (s)	0.338	0.34	0.339
QT interval std.(s)	0.009	0.059	0.008
ST level mean (mV)	-0.035	-0.213	0.11
ST level std. (mV)	0.027	0.038	0.021
ISO level mean (mV)	-0.294	0.21	-0.326
ISO level std.(mV)	0.079	0.025	0.062

**Fig 9:**
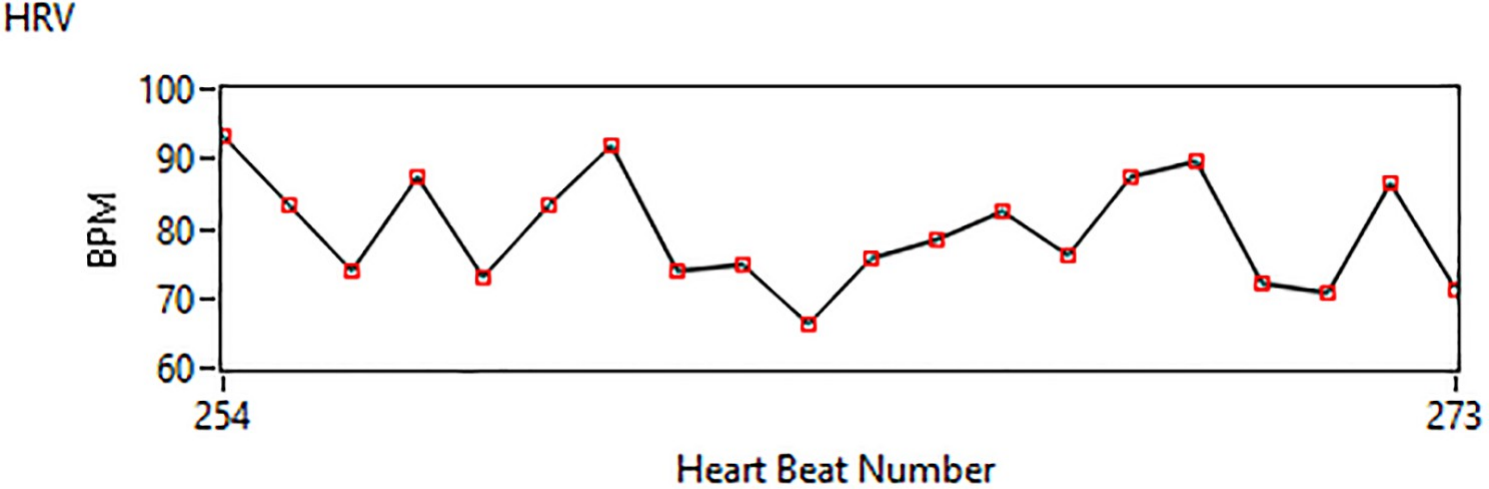
Measurement of HRV.

The next variable that is part of our analysis is heart rate variability (HRV). This is a variation in time intervals between the consecutive heartbeats. HRV is regulated and controlled by the basic part of our nervous system called the autonomic nervous system (ANS). An abnormal HRV pattern leads to life-threatening cardiac diseases such as arrhythmia. HRV can be affected by other factors, including aging and gender. Fig 9 reflects the heart rate number under normal conditions.

### Wavelet scattering transform

ECG time-series signals usually require higher frequency resolution than time resolution because they have a low-frequency component. However, a high time resolution is required for high-frequency components in ECG because they vary quickly with time. Therefore, a multi-resolution analysis method would analyze an ECG signal precisely if the ECG signal comprises both high- and low-frequency components.

**Fig 10:**
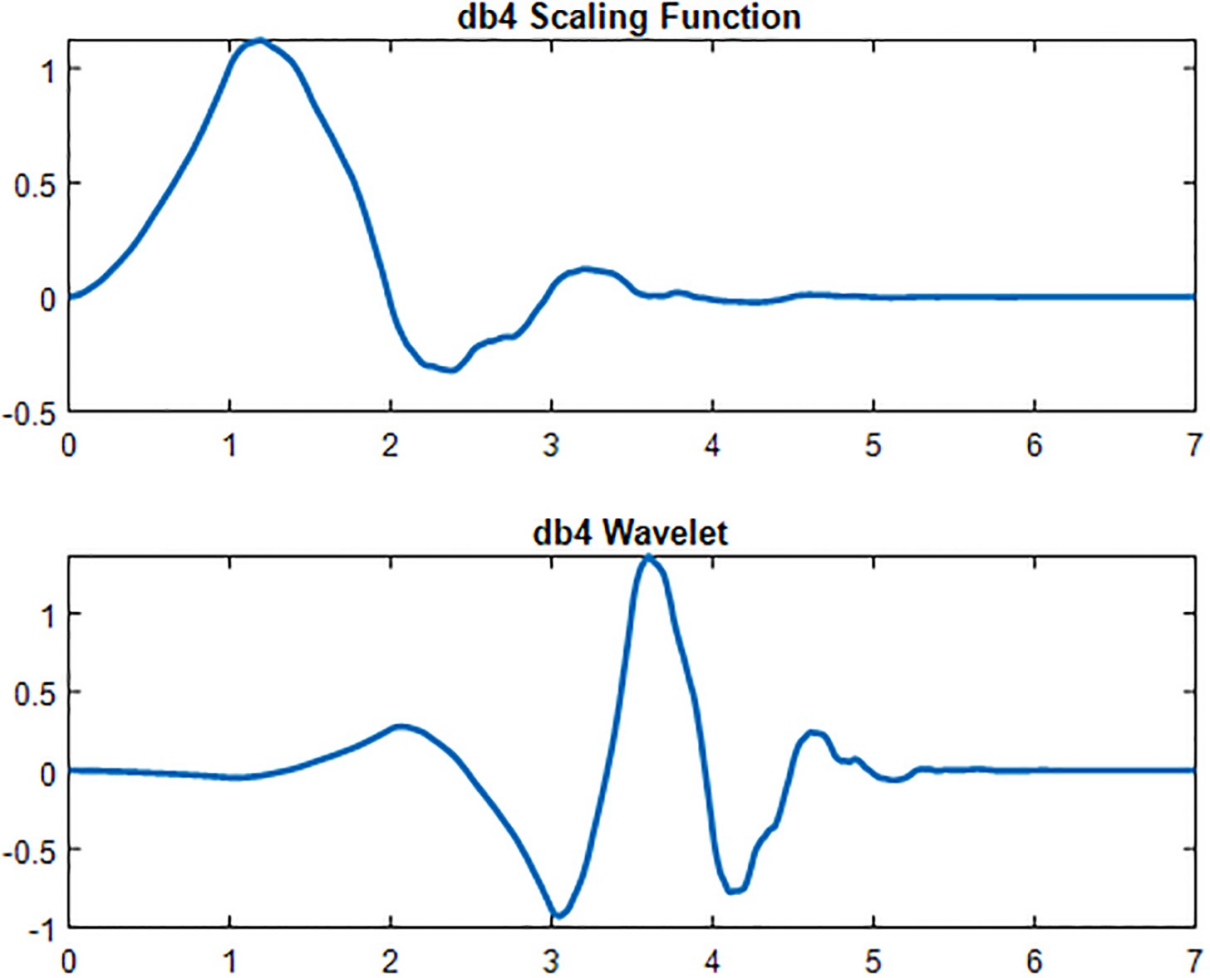
Wavelet transform and scaling function.

In wavelet scattering, the propagation of data is done by a series of wavelet transforms as shown in Fig 10, nonlinearities, and averaging. Therefore, it produces low-variance representations of time series.

Wavelet time scattering yields signal representations insensitive to shifts in the input signal without sacrificing class discriminability. The selection of wavelet filter type has a significant effect on the overall de-noising performance of the proposed method. The wavelet transform has made the scale discrete by using the Daubechies-4 (db4) wavelets of wavelet filters since the data is identical with an in-variance scale 15. The selection of db4 Wavelet as a measuring parameter is because of the resemblance between its scaling function and the shape of the actual ECG signal.

### Anomaly detection through LSTM-AE

**Fig 11:**
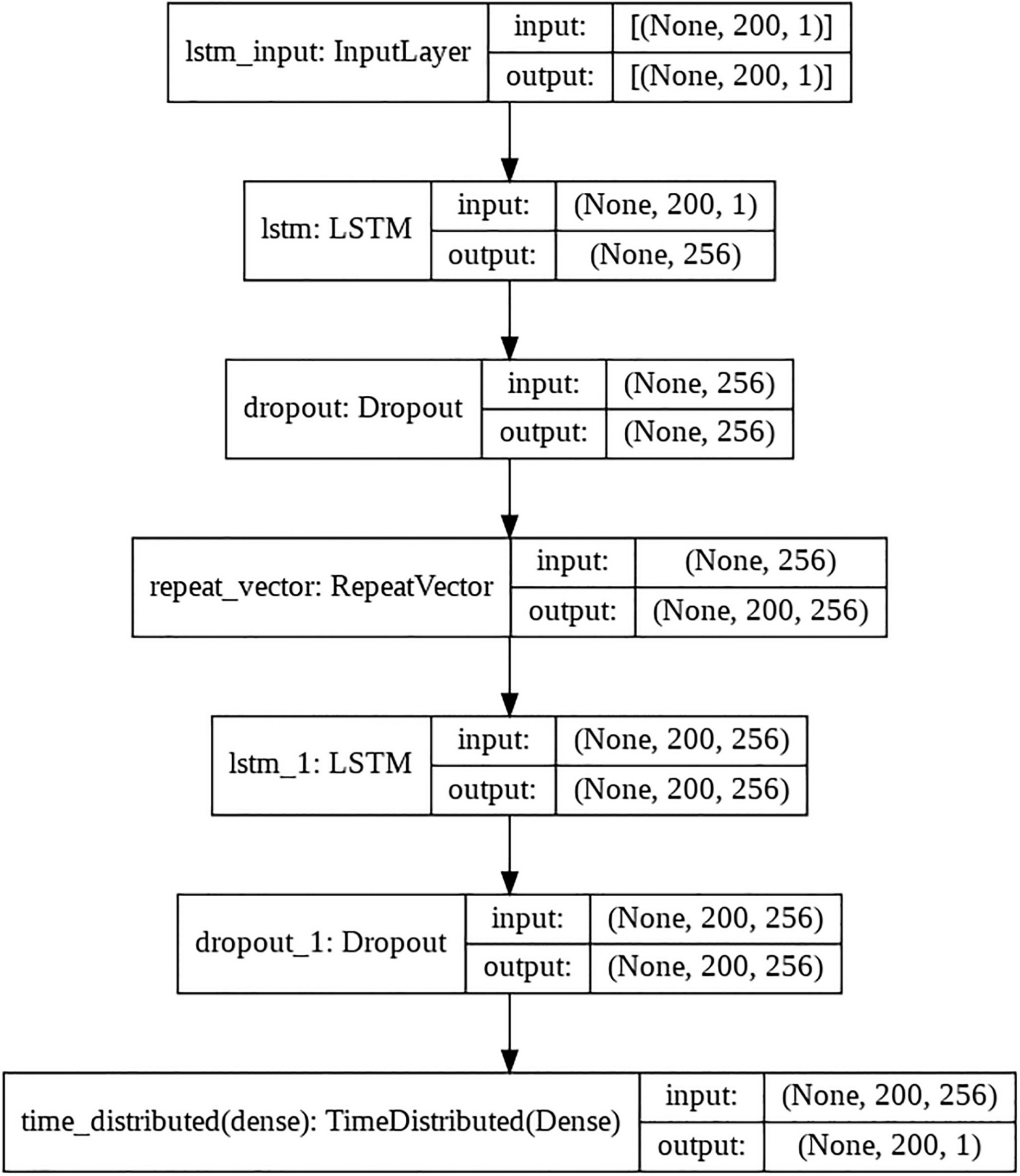
Model summary.

The extended part of this research work contributes to the system by making the design intelligent through end-to-end anomaly detection and classification of the ECG signals. An LSTM-AE-based deep neural network is implemented to detect anomalies in acquired ECG signals. To augment the training dataset, a 1 x 65536 samples long signal is divided into 200 sample chunks and fed to AE for achieving bettering accuracy. The model is implemented in Keras, and the model summary is shown in Fig 11. The model used in the classifier has a simple structure of one LSTM layer with 256 followed by a dropout layer with a rate of 0.2. Then, a repeat vector followed by the decoder level of the LSTM layer with 256 and a time-distributed layer is applied. Adam optimizer is used with mean absolute error (MAE) loss. An average MAE loss of 0.0072 for normal signals is achieved, and 0.078 is achieved for anomalous signals.

**Fig 12:**
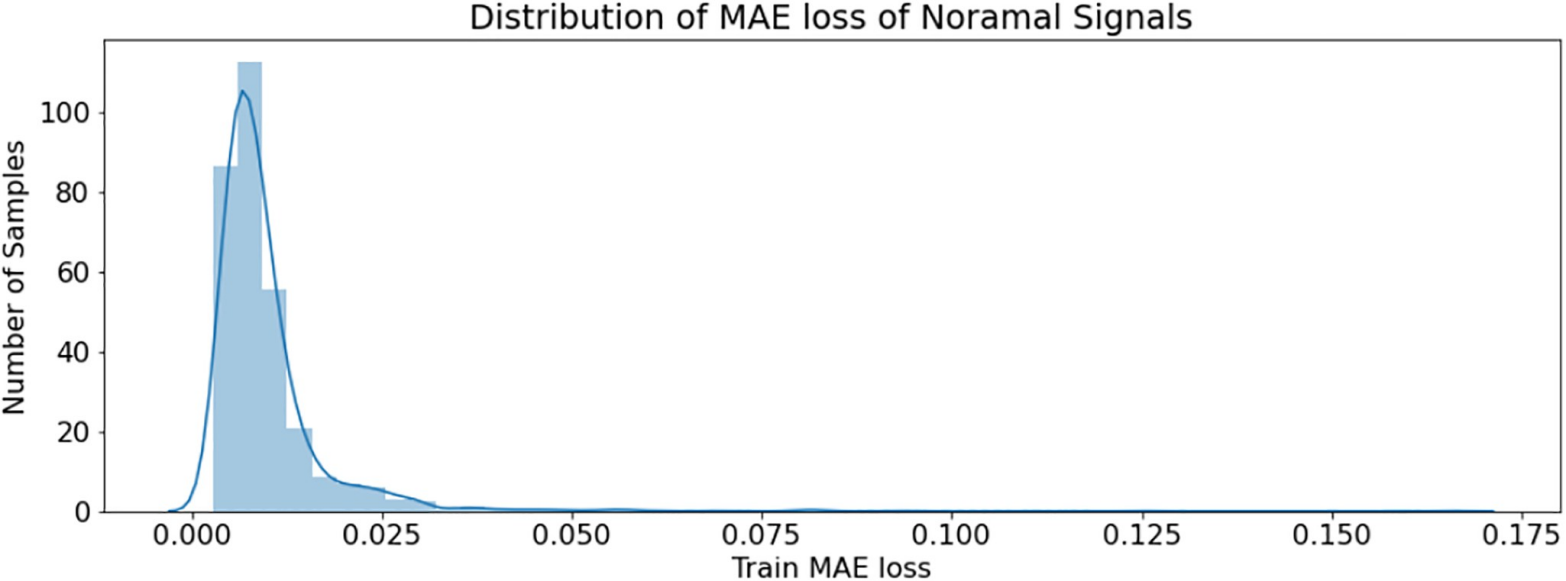
Distribution of normal signals loss.

**Fig 13:**
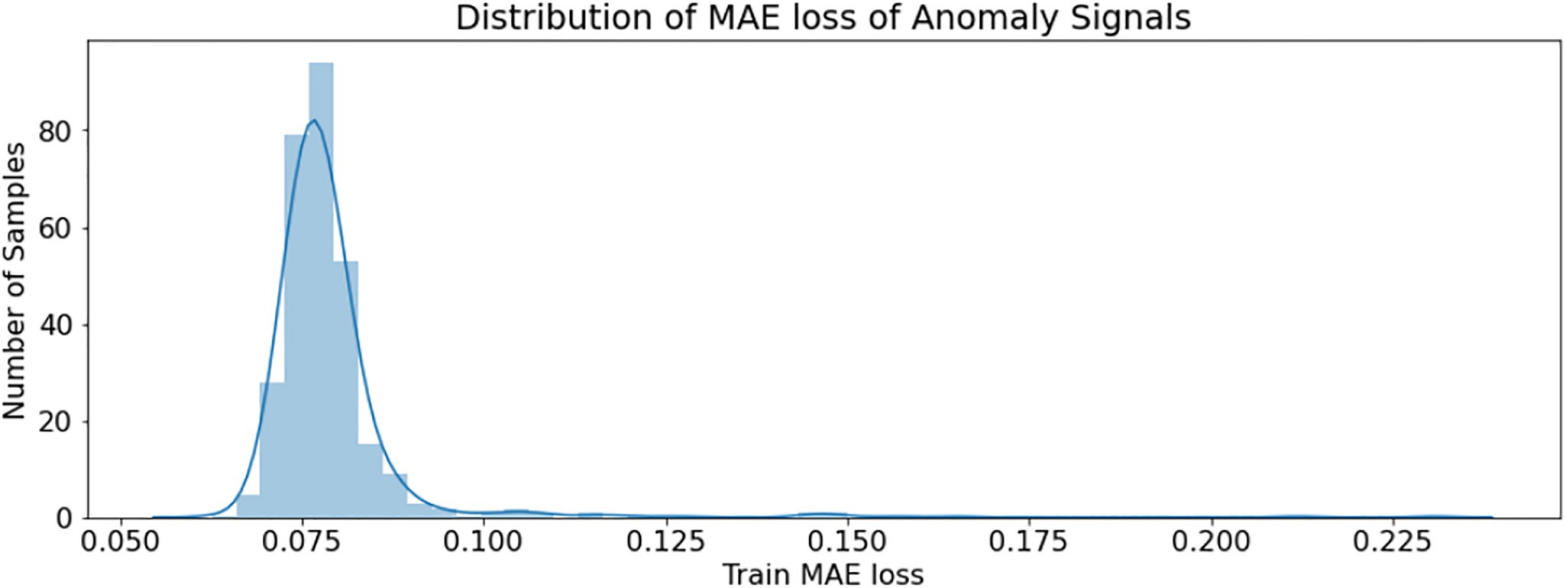
Distribution of anomaly signals Loss.

**Fig 14:**
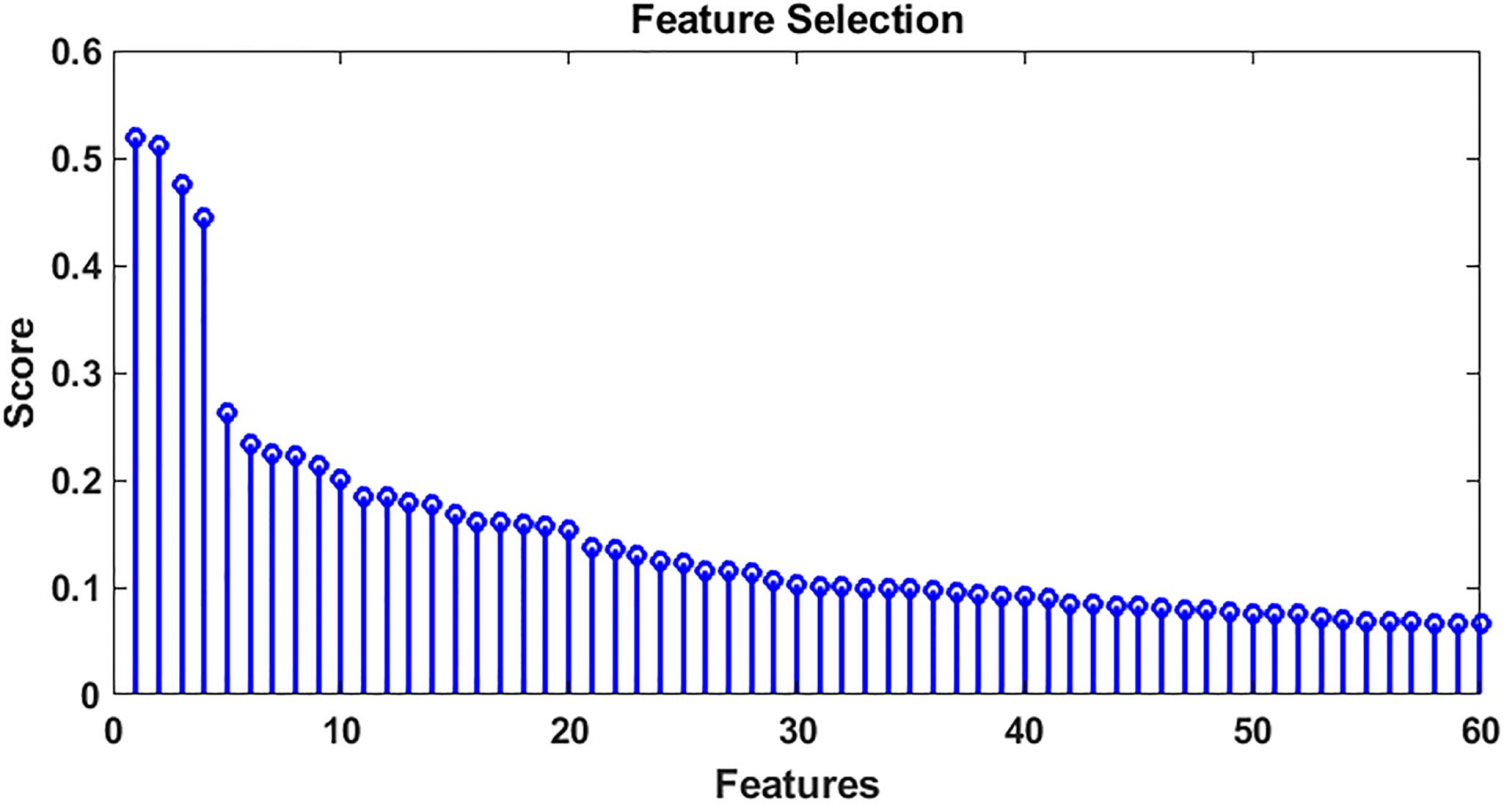
Reconstruction error of normal & anomaly signals.

Fig 12 shows the distribution of normal signal Mae loss, while Fig 13 shows the distribution of anomaly signal loss. It is shown that 98% of the normal signal loss distribution is under 0.05, while the distribution of anomaly signal Mae loss is after 0.07. Therefore, anomalies are accurately detected by setting the maximum loss threshold of 0.068. The model’s ability to reconstruct normal signals and anomaly signals is shown in Fig 14. The normal signals model has much less reconstruction error loss than anomaly signals by learning/mapping the dependencies present in the input signal. The first row of signal plots in Fig 14 is for the normal signal compared with the reconstructed signal (red), and the second row of signal plots shows the anomaly signal compared with the reconstructed signal (red). It is clear from the graphs that the anomaly reconstruction error is almost 10 times greater than the normal signal reconstruction error loss. By setting reconstructed error thresholds of 0.02 in terms of MAE loss, 98% accuracy is achieved for anomaly detection. We have implemented an automatic detection approach to detect anomalies using an LSTM-AE. This deep learning-based approach enables the discovery of constraints involving long-term nonlinear associations among multivariate time-series data records and attributes.

### ECG classification results

**Fig 15:**
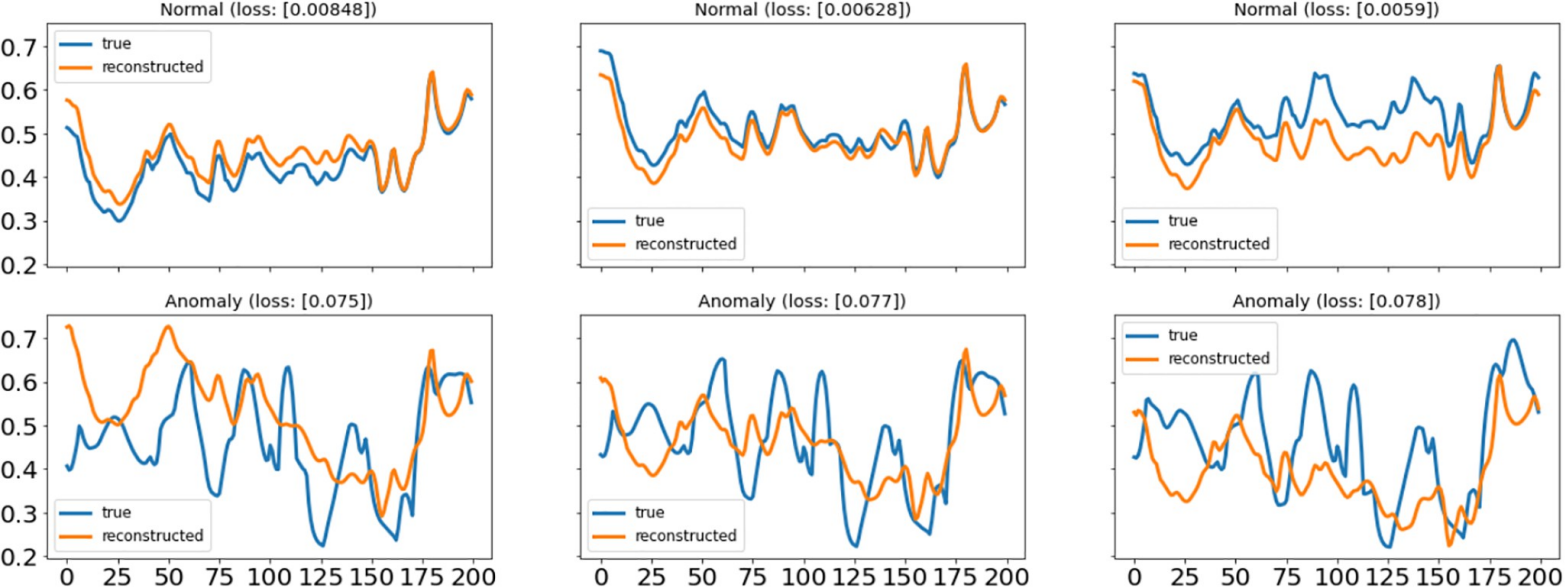
Feature selection.

A wavelet scattering filter bank is constructed giving the length of the signal, sampling frequency, quality factor of [8, 1], and default invariance scale. We extracted the features from Fig 15, using the function feature matrix, reducing down to a feature set of 499 by 8, which is a 95% reduction in the size of features. We separated 113 samples for training, and 49 samples were excluded from ECG samples for testing and randomly selected and computed the feature matrix of all of these data for both the test and training samples. The output of the training data feature set is 499x8x113. The wavelet scattering transform is critically down-sampled in time based on the bandwidth of the scaling function. In this case, this results in 8-time windows for each of the 499 scattering paths. The data were then trained by implementing 25 machine learning algorithms, including KNN, SVM, ensemble subspace discriminant, and subspace KNN, which had higher accuracy, as shown in Table 2. While evaluating this trained model on the test data, the accuracy of the predicted class will be compared with the true class accuracy. We obtained the classification results based on different numbers of feature sets.

**Table 2:** Classification accuracy of ML algorithms on different feature sets.

Selected features	Accuracy with 499 features		Accuracy with 60 features		Accuracy with 20 features	
Classification model	Training (%)	Testing (%)	Training (%)	Testing (%)	Training (%)	Testing (%)
Trees Fine	97.6	94.89	97.3	93.36	97.6	93.36
Trees Medium	97.6	94.89	97.3	93.36	94.6	93.36
Trees Coarse	89.6	90.81	89.2	86.48	89.0	86.48
Linear Discriminant	99.1	82.9	99.7	95.91	95.9	91.58
Naïve Bayes-Gaussian	83.3	82.3	86.8	86.98	86.2	85.96
Naïve Bayes-Kernel	93.4	85.2	97.6	96.17	98.1	94.64
SVM-Linear	96.8	96.17	98.8	97.19	97.9	92.60
SVM-Quadratic	99.8	94.13	99.8	96.42	99.9	97.19
SVM-Cubic	99.9	99.23	100	97.19	99.7	95.91
SVM-Fine Gaussian	83.1	63.7	95.5	67.09	86.2	72.19
SVM-Medium Gaussian	96.3	95.4	99.8	97.9	99.3	97.19
SVM-Coarse Gaussian	82	80.35	87.4	85.20	88.6	86.73
KNN-Fine	100	94.39	100	96.17	99.9	94.64
KNN-Medium	95.7	91.07	97.9	93.87	98.8	92.85
KNN-Coarse	75.9	78.06	85	89.28	91.6	92.60
KNN-Cosine	95.2	88.01	96.8	95.92	97.7	95.15
KNN-Cubic	94.5	88.26	96.9	92.60	97.7	89.54
KNN-Weighted	99	93.10	99.3	93.87	99.4	92.60
Ensemble-Boosted Trees	59.3	59.18	59.3	59.18	59.3	59.18
Ensemble-Bagged Trees	97.5	97.4	96.6	92.09	96.7	89.28
Ensemble-Subspace Dis.	99.8	95.15	97.6	96.42	91.3	87.24
Ensemble-Subspace KNN	100	90.56	99.7	95.91	99.6	98.04
Ensemble-RUSBoosted Trees	98.3	94.64	98.1	95.15	99.0	95.15
LSTM	100	100	-	-	-	-

We trained the raw signal by inputting all 499 features, and we observed SVM fine, cubic, medium Gaussian, and ensemble subspace discriminant with prominent testing accuracy.

For improved feature selection, we used the “fscmrmr” function [67], which is an automatic feature selection algorithm that ranks features for classification using the minimum redundancy maximum relevance algorithm. It calculates the scores for all the training data sets and evaluates them on what features have a greater impact and which features have less impact.

If we plot the first 60 features as shown in Fig 14, feature number one has a very high score, then the second-highest score is feature number 141. Third highest, 395, 73, and so on. Therefore, for the next training, we used the top 20 feature sets, not using the entire 499 feature set. Similarly, when there are only 20 features to be used for training, there are fewer feature sets, so the training would be very quick. We reduced the feature set to 60 samples to include only the most important features, and we observed improvements in the accuracy of most SVM-, KNN- and ensemble-based ML algorithms. By reducing the feature set up to the 20 most important samples, we were able to obtain testing accuracy up to 98%.

The three classes that we need to build a model to classify the signals are ARR, CHF, and NSR. In each class, we have approximately 96 signals for the first class and 30 signals for the second and third classes, which is the NSR, with 36 signals. Each signal is approximately 65,536 samples long. Now, the goal of this research is to find an optimal algorithm that can classify ECG signals into these three distinct categories most accurately. Therefore, applying ML techniques can reduce the dimensions of the signal automatically and extract the features while losing information about the signal itself.

The main idea is to reduce the signal dimensions while preserving the information. Therefore, DL algorithms have been implemented so that the signal does not lose any information during training. The sample signal we used earlier had 65,536 To predict class labels, the network ends with a fully connected layer, a softmax layer, and a classification output layer. Through the dropout function, some hidden outputs do not influence the training of the model. However, during testing the network dropout function will be turned off and all the hidden neurons output will affect the testing procedure. In this paper one dropout layer is used during training of the LSTM network model. We can directly feed in the signal to the DL-based LSTM network, and under a few situations, instead of feeding the signals directly to LSTM networks, we can perform feature extraction before feeding the signals into the LSTM network, if required. We can train our LSTM networks on those features to build our model in situations where feeding raw data directly into LSTMs does not work, if we have fewer data to begin with, which is typically the case for many AI-based problems, or in situations where data augmentation can be very challenging.

The samples, and the features that were extracted automatically had 499 x 8 features. Therefore, there are approximately 4,000 features in the data set. This shows a 95% reduction in the size of the features compared to the original signal. We have taken all the features, which is 499 x 8, by taking that as the whole matrix and then trained the LSTM network. We built a small LSTM network using Matlab 2020’s deep network designer app., Table 3 shows the detail of the network with the total number of hidden units, number of layers, and other details.

**Table 3:** Optimized network structure.

Sr. no.	Layer detail	Type	Activation	Learnables
1	Sequence input with 499 dimensions	Sequence input	499	-
2	LSTM with 300 hidden units	LSTM	300	Inputweights 1200*…, RecurrentWei 1200*…
3	3 fully connected layers	Fully connected	3	Weights 3*300: Bias 3*1
4	Softmax	Softmax	3	-
5	Class output	Classification output	-	-

We trained the network by hyper-parameters tuning using Matlab 2020 Experiment manager. The experiment manager trained the network defined by the setup. By default, one trial is run at a time, and combinations are made in each trial. The parameters such as the activation functions are selected by default where the state activation function is “tanh” and the gate activation function is “sigmoid”. The parameters for training the network were selected after finding the optimized parameters. Table 4 shows the values and evaluation of parameters such as initial learning rate, mini-batch size, optimizer, maximum epoch size, and “last” as the output mode after hyper-parameter optimization, giving 100% classification accuracy. The input side is 499 because they are the number of rows for every signal.

**Table 4:** Hyper-parameters selection for model training and validation.

Optimizer	Initial learn-rate	No. of epochs	Mini batch-size	Training accuracy	Training loss	Val. accuracy	Val. loss
Adam	0.01	150	1000	100.000	0.0003	97.8776	0.2184
**Adam**	**0.1**	**150**	**1000**	**100.000**	**0.0170**	**100.000**	**0.0015**
Adam	0.01	200	1000	100.000	0.0003	91.8367	0.5997
Adam	0.1	200	1000	98.2301	0.0537	95.9184	0.1574
Adam	0.01	150	1500	100.000	0.0003	97.8776	0.2184
Adam	0.1	150	1500	100.000	0.0170	100.000	0.0015
Adam	0.01	200	1500	100.000	0.0003	91.8367	0.5997
Adam	0.1	200	1500	98.2301	0.0537	95.9184	0.1574
Sgdm	0.01	150	1000	59.2920	0.8774	59.1837	0.8813
Sgdm	0.1	150	1000	90.2655	0.2548	73.4694	0.9484
Sgdm	0.01	200	1000	61.0619	0.8289	59.1837	0.8372
Sgdm	0.1	200	1000	58.4071	1.0298	55.1020	1.1230
Sgdm	0.01	150	1500	59.2920	0.8774	59.1837	0.8813
Sgdm	0.1	150	1500	90.2655	0.2548	73.4694	0.9484
Sgdm	0.01	200	1500	61.0619	0.8289	59.1837	0.8372
Sgdm	0.1	200	1500	58.4071	1.0298	55.1020	1.1230
RMSprop	0.01	150	1000	91.1504	0.2191	81.6327	0.6995
RMSprop	0.1	150	1000	92.0354	0.2714	85.7143	0.3917
RMSprop	0.01	200	1000	64.6018	1.4296	81.6327	0.6995
RMSprop	0.1	200	1000	94.6903	0.1523	87.7551	0.4946
RMSprop	0.01	150	1500	91.1504	0.2191	81.6327	0.6995
RMSprop	0.1	150	1500	92.0354	0.2714	85.7143	0.3917
RMSprop	0.01	200	1500	64.6018	1.4296	81.6327	0.6995
RMSprop	0.1	200	1500	94.6903	0.1523	87.7551	0.4946

In Table 4 all the combinations of hyper-parameters are given. Among them, we have selected the parameters having the best validation/testing accuracy. The selected parameters are adam as an optimizer, 150 epochs, and 1000 mini-batch size gives 100% validation/testing accuracy. Different values for mini-batch size were tested ranging from 800-1500 and it showed no major difference in performance with a minor difference in training time. Therefore, we used the mini-batch size value as 1000.

**Fig 16:**
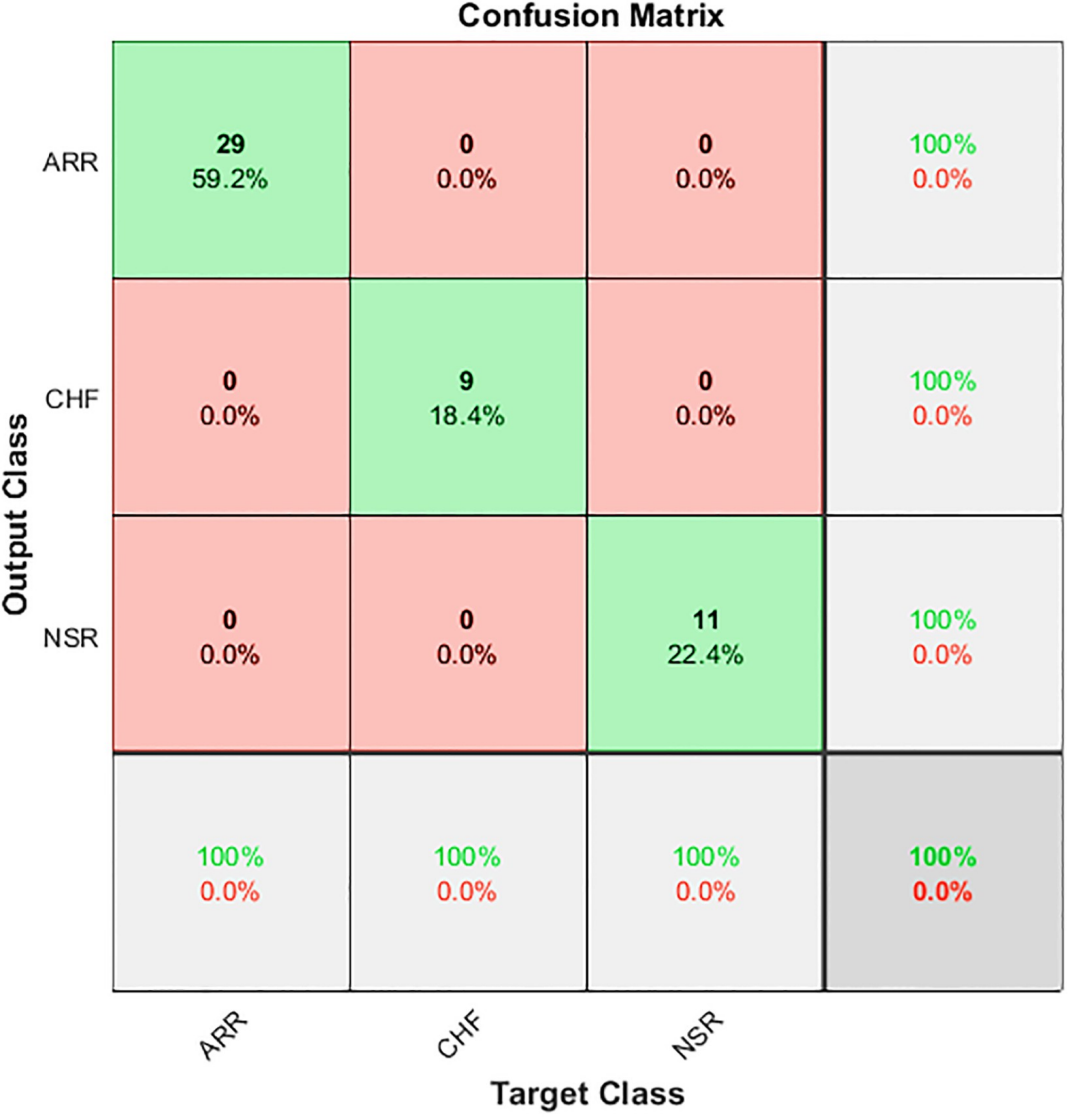
LSTM confusion matrix for ECG classification.

The LSTM network is trained quickly, approx. in 45 seconds. To evaluate the LSTM model, we extracted all the features from the test signals. This model yields 100% accuracy, as shown in Fig 16. Thus, LSTM has proven to be the optimal technique without the limitation of losing information and being accessible to noise due to reduction in the feature set, being a quick solution for raw 1D ECG data classification.

**Fig 17:**
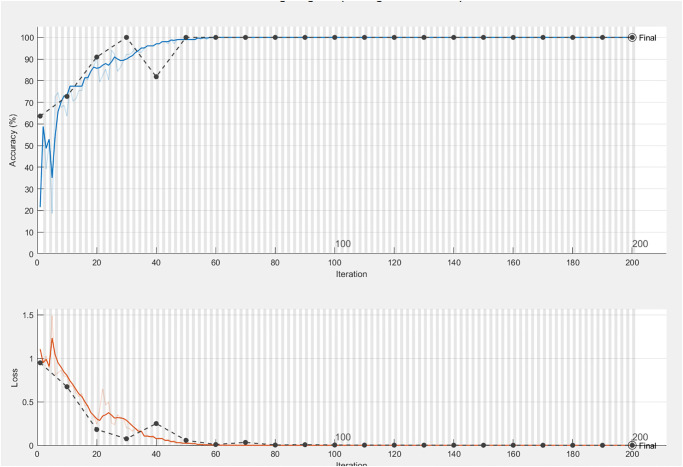
Training & validation accuracy/loss curves.

In order to validate our algorithm, the accuracy and loss curves have been plotted in Fig 17. This figure shows the accuracy and loss curves of training and validation data. A maximum accuracy of 100% has been obtained on the 50th iteration. Similarly, the loss curve shows a continuous decay after the 40th iteration.

**Fig 18:**
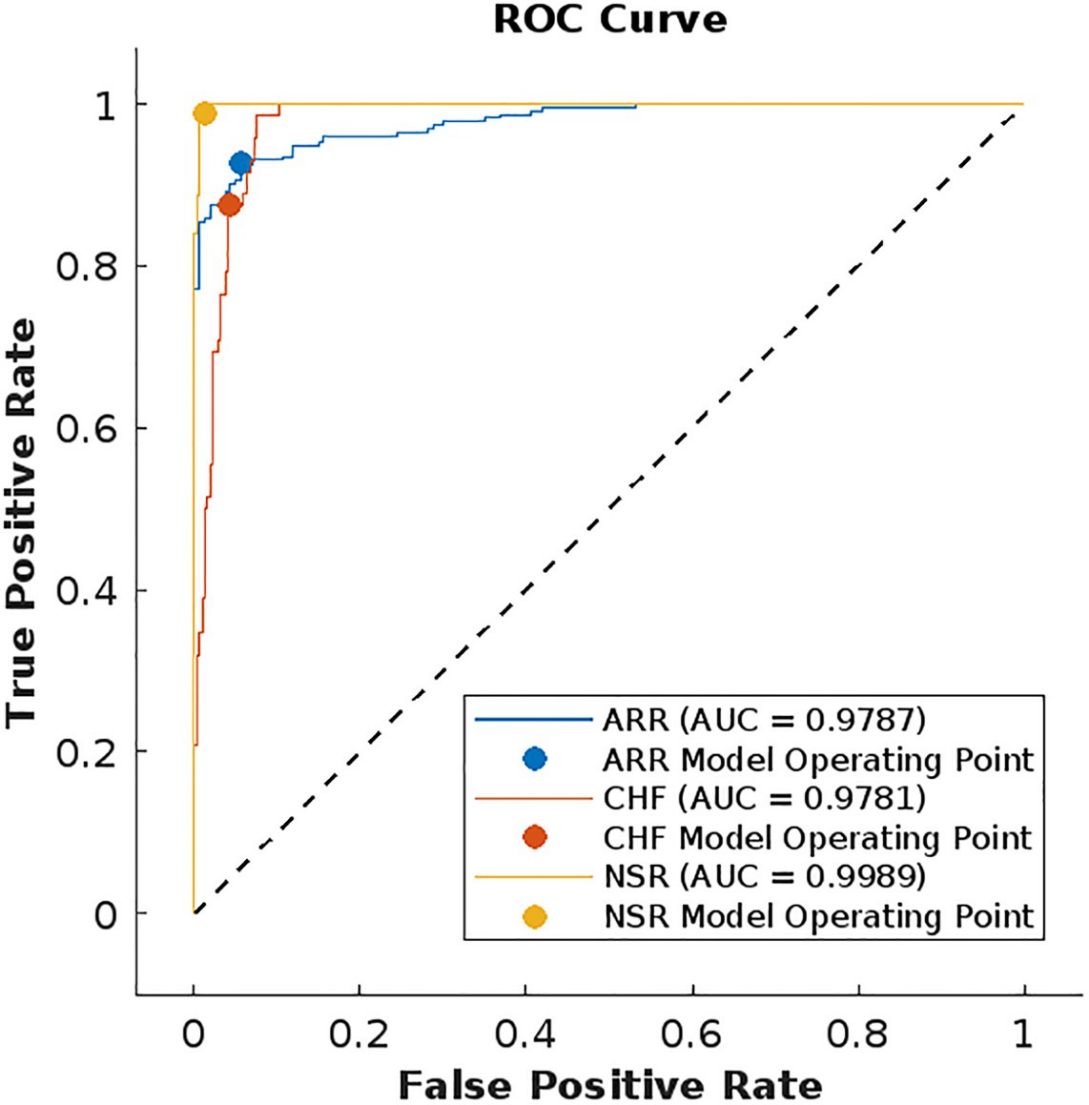
ROC curves for each class using KNN-weighted.

**Fig 19:**
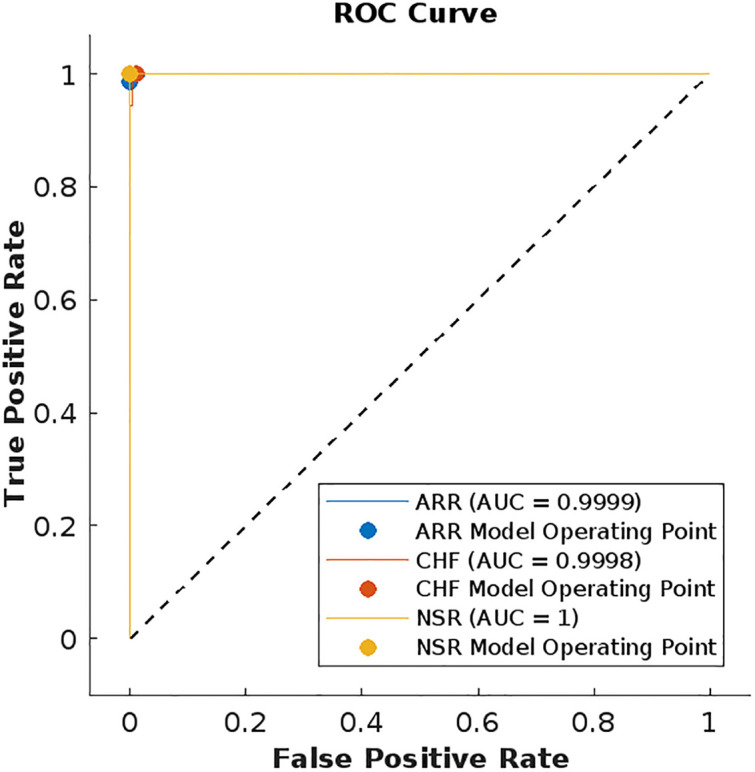
ROC curves for each class using SVM-cubic.

**Fig 20:**
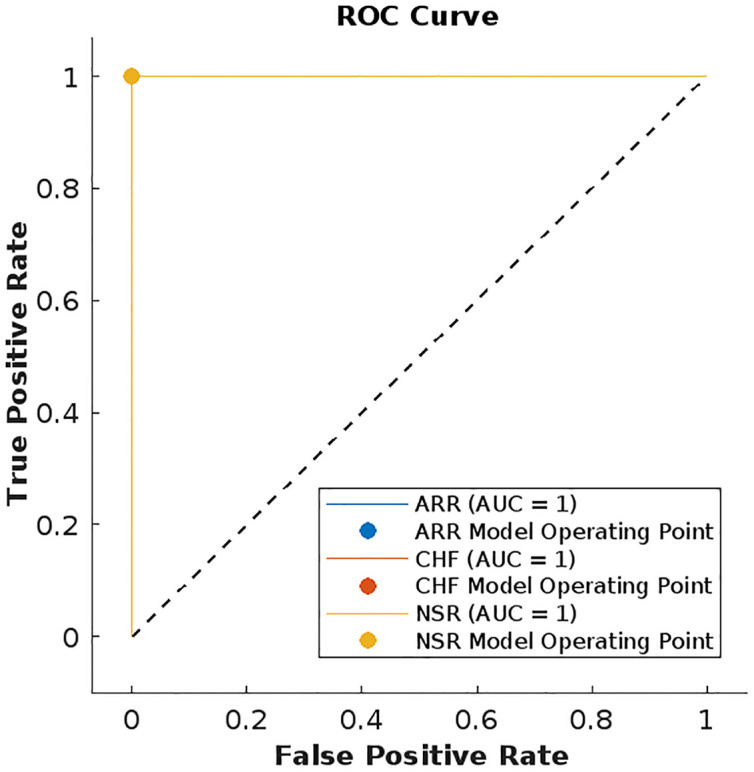
ROC curves for each class using LSTM.

We have also plotted the receiver operating characteristic (ROC) curves for evaluating the performance of our model with other ML models. The models in Table 2 having better accuracy rates are SVM cubic and KNN weighed, therefore their ROC curves are shown in Figs 18 and 19. ROC curves show the relationship between the true positive rate and false positive rate and it shows the quality of a classifier ranging [0, 1]. The area under the curve (AUC) in the figures shows the performance level of the classifier. The ROC curve of the LSTM model in Fig 20 shows the highest quality.

**Fig 21:**
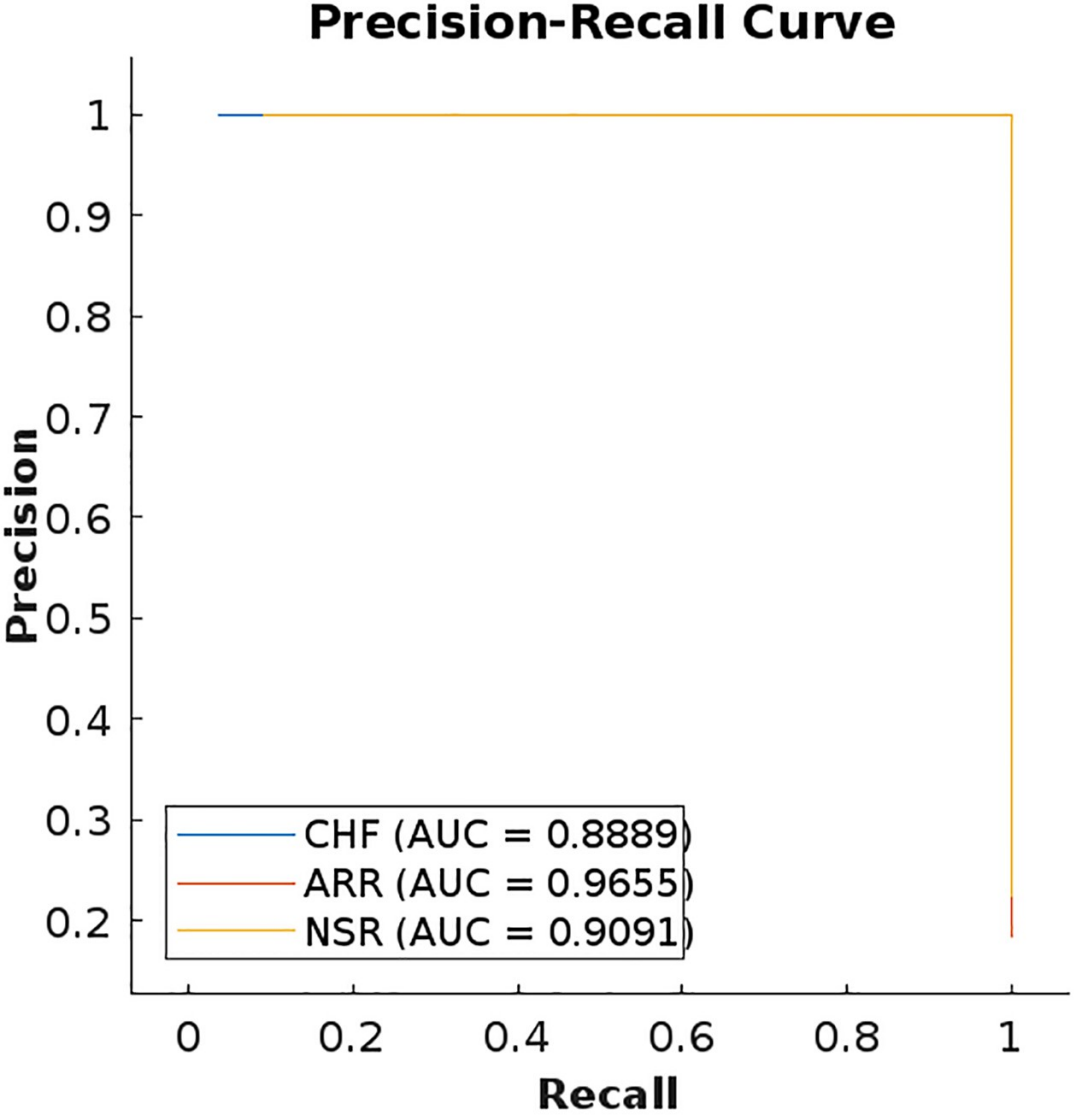
Precision-recall curve (PRC) of LSTM.

For the final evaluation of our implemented model, we have plotted the precision-recall curve (PRC). The PRC has emerged as a good indicator of a classifier’s performance. A PRC of LSTM is shown in Fig 21 which shows a high area under the curve for both recall and precision values. A high precision is associated with a low false positive rate, similarly, a high recall is linked to a low false negative rate.

Out of all evaluated classifiers in Table 2, the classifiers having better testing accuracy have been evaluated using other metrics including precision, recall, and F1 score. On the basis of all these parameters, Table 5 incorporates the comparison.

**Table 5:** Performance evaluation of model classifiers.

Classifier Models	Precision	Recall	F1 Score
SVM Cubic	0.9867	0.9957	0.9912
KNN Weighted	0.9233	0.9492	0.9361
Ensemble Bagged Tree	0.9341	0.9656	0.9496
Our method (LSTM)	1.000	1.000	1.000

Table 6 summarises the testing evaluation scores of classifiers with respect to each class individually. According to that our method outclasses other methods with 100% precision, recall, and F1 score for each class.

**Table 6:** Testing metric evaluation of each class.

Classifier	Classes	Precision	Recall	F1 Score
SVM Cubic	ARR	100	98.27	99.13
	CHF	94.74	100	97.30
	NSR	100	100	100
KNN Weighted	ARR	97.74	93.10	95.36
	CHF	84.62	91.67	88
	NSR	94.63	100	97.24
Ensemble Bagged Tree	ARR	98.65	94.40	96.48
	CHF	85.71	100	92.31
	NSR	98.84	96.60	97.70
LSTM (Our method)	ARR	100	100	100
	CHF	100	100	100
	NSR	100	100	100

In Table 7, our method has been compared with existing literature and the results show that our approach outclasses others in accuracy, precision, recall, and F1 score.

**Table 7:** Performance evaluation of our approach with-respect-to literature.

Sr.no	Literature	Accuracy	Precision	Recall	F1 Score
1	[51]	89.82	-	-	-
2	[68]	98.75	-	-	-
3	[69]	98.74	-	-	68.76
4	[70]	-	98.41	-	-
5	[71]	99.37	96.73	-	95.77
6	[72]	99.73	93.60	-	91.80
7	[45]	97.42	-	-	-
8	[48]	97.75	-	-	86.0
9	[49]	99.52	-	-	-
10	Our Method	100.0	100.0	100.0	100.0

## Conclusion

In this work, an AWS cloud-based framework has been designed and implemented for automatic anomaly detection and classification of 1D ECG signals. The anomaly detection of raw ECG signals has been implemented using an LSTM auto-encoder. The results show a reconstructed error threshold of 0.02 in terms of MAE loss with 98% accuracy for anomaly detection. The proposed system is effective and time-efficient for large datasets. The wavelet time scattering feature extraction method has achieved 95% signal reduction to increase the real-time processing speed. In addition to anomaly detection, this work provides an extensive comparative study of state-of-the-art techniques to identify the optimum solution for the classification of ECG signals.

An improved feature set has been developed in this study by combining the carefully crafting hand-engineered features with automatically detected features through AI-based algorithms to make the system more effective.

The comparative evaluation has shown that our approach, the deep-learning-based algorithm LSTM has shown 100% testing accuracy, F1 score, Precision and Recall for multi-class ECG signal giving in the raw 1D ECG data with 95% reduction in signal length to make the proposed system efficient.

In the future, we would work on improving the automatic ECG anomaly detection and classification system, by making it patient-specific to challenge the classifier for temporal and morphological variations in different patients.

The framework can be extended to real-time analysis platforms such as spark streaming or Apache Kafka open-source solutions.

The limitation of this study is that the proposed system was not validated against the hospital patient data. We have only used the publicly available dataset.
